# QTL Map Meets Population Genomics: An Application to Rice

**DOI:** 10.1371/journal.pone.0083720

**Published:** 2013-12-23

**Authors:** Jeffrey A. Fawcett, Tomoyuki Kado, Eriko Sasaki, Shohei Takuno, Kentaro Yoshida, Ryuichi P. Sugino, Shunichi Kosugi, Satoshi Natsume, Chikako Mitsuoka, Aiko Uemura, Hiroki Takagi, Akira Abe, Takashige Ishii, Ryohei Terauchi, Hideki Innan

**Affiliations:** 1 Graduate University for Advanced Studies, Hayama, Kanagawa, Japan; 2 Iwate Biotechnology Research Center, Kitakami, Iwate, Japan; 3 Iwate Agricultural Research Center, Kitakami, Iwate, Japan; 4 Faculty of Agriculture, Kobe University, Kobe, Japan; National Rice Research Center, United States of America

## Abstract

Genes involved in the transition from wild to cultivated crop species should be of great agronomic importance. Population genomic approaches utilizing genome resequencing data have been recently applied for this purpose, although it only reports a large list of candidate genes with no biological information. Here, by resequencing more than 30 genomes altogether of wild rice *Oryza rufipogon* and cultivated rice *O. sativa*, we identified a number of regions with clear footprints of selection during the domestication process. We then focused on identifying candidate domestication genes in these regions by utilizing the wealth of QTL information in rice. We were able to identify a number of interesting candidates such as transcription factors that should control key domestication traits such as shattering, awn length, and seed dormancy. Other candidates include those that might have been related to the improvement of grain quality and those that might have been involved in the local adaptation to dry conditions and colder environments. Our study shows that population genomic approaches and QTL mapping information can be used together to identify genes that might be of agronomic importance.

## Introduction

Improving the yield and quality of crops is one of the biggest challenges in plant biology, and efforts are being made to identify genes underlying phenotypic variation that could be utilized for crop improvement [1]. One of the most popular approaches to search for such genes has been the mapping of quantitative trait loci (QTL) [2]. However, although a huge amount of QTL information has accumulated in various species, such a rough marker-based survey rarely provides enough information to actually identify the gene responsible for the trait of interest. In addition, a top-down approach starting from a QTL or candidate gene of interest would be biased towards prior knowledge [3], [4]. An alternative approach that is recently gaining popularity is a bottom-up population genomics approach where one screens for genes or regions that differentiate cultivated species from wild species, or certain cultivars from others based on genomic polymorphism data [5], [6], [7], [8], [9]. Although this approach can identify multiple relatively small regions (compared to QTL mapping), these regions can still contain a large number of genes, most which are not the actual target gene, due to genetic hitchhiking. Thus, the identification of such genes of interest remains a challenge. Here, we combine the population genomics approach with QTL mapping information to identify candidate genes selected during the domestication of rice that should be of agronomic importance.

**Figure 1:**
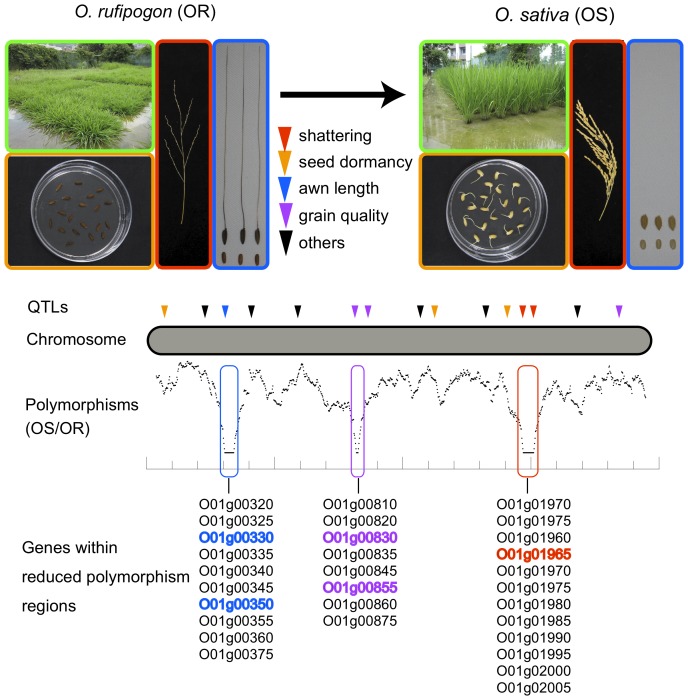
Strategy to identify genes targeted by selection during the domestication process. A hypothetical example of a chromosome with polymorphism data is shown. The domestication process of *O. rufipogon* to *O. sativa* should have resulted in changes in traits such as shattering, seed dormancy, awn length, and grain quality, among others, and the QTLs related to these traits have been roughly mapped on to the genome (shown by triangles). In addition, genomic regions that contain domestication genes should show reduced levels of polymorphisms due to selective sweep. We reasoned that these regions should overlap with the domestication related QTLs, and contain genes with functions related to such QTLs (those indicated by colors).

As illustrated in Figure 1, substantial morphological and physiological differences exist between the cultivated rice *Oryza sativa* and the wild rice *O. rufipogon* as a result of strong artificial selection on certain alleles during the process of domestication. Regions containing alleles that were fixed by selection during the domestication process are expected to show reduction of polymorphisms. This is because other genetic variants in neighboring regions are swept out by the hitchhiking effect [10]. At the same time, cultivated species often show reduction in the genome-wide genetic variation due to the bottleneck in the initial phase of domestication. It is nevertheless possible to identify regions of selective sweeps because these regions should show a reduction of polymorphisms that is significantly greater than what would be expected by the bottleneck effect alone. These regions can contain tens of or even over a hundred genes, especially in selfing species such as rice [8], [11], [12]. Thus, it is still necessary to further narrow down the candidate genes within each region based on some other information before experimentally testing each gene. For instance, He et al. [7] chose to report genes within low diversity regions that have at least one nonsynonymous substitution distinguishing the cultivated species from the wild species. However, this could be misleading because the target of selection could be various other kinds of mutations [3], [13], [14]. Also, the relevant mutations could fall in poorly sequenced/assembled regions and go undetected.

In this study, we first resequenced more than 30 genomes altogether of *O. rufipogon* Griff and the two subspecies *indica* and *japonica* of *O. sativa* L., and then identified regions where variation is reduced due to directional selection. To gain further insight into the genes targeted during the domestication process, we took advantage of the wealth of QTL information in rice [15]. We searched for QTLs that map to each region, particularly focusing on QTLs related to shattering, seed dormancy, awn length, and grain quality; traits that are clearly different between cultivated and wild rice. With the aim to assist future studies, we created a list including all QTLs that map to each selective sweep region. We also created a list with all genes within each region, together with information of their expression pattern [16], and a list of genes within each region that contain sequence variants that are fixed in *O. sativa*. The information we have gathered allows us to make more meaningful speculation on the candidate genes, which should motivate further empirical verifications. In particular, we discuss a number of interesting candidate genes that were likely targeted by selection during the domestication of *O. sativa*, and also genes likely involved in the differentiation between *indica* and *japonica*, and between temperate and tropical *japonica*.

## Results

We will first overview the genome-wide pattern of SNPs revealed by resequencing over 30 strains of *O. sativa* ssp. *indica*, ssp. *japonica* and *O. rufipogon*. After that, we will describe the identification of candidate regions of various modes of domestication selection by applying population genomic techniques. This requires precise understanding of the pattern of SNPs, such as the levels of polymorphism and linkage disequilibrium.

### Sequencing genomes

We sequenced the entire genomes of 12 *O. sativa* ssp. *indica*, 10 ssp. *japonica,* and 10 *O. rufipogon* accessions by using the Illumina next-generation sequencing platform. These accessions were sampled to cover the major geographic ranges of wild and cultivated rice (Table 1). After sampling, they were inbred for several generations so that most genomic regions are expected to be homozygous. In total, we obtained 1.62×10^9^ paired-end reads of 75-bp nucleotides ( =  243.0 Gb). *Japonica* accessions were sequenced to an average of x18 depth and 94% coverage, and *indica* accessions to an average of x15 depth and 88% coverage. *O. rufipogon* accessions were sequenced to a higher depth (x21 on average) as they are more diverged from the reference genome (Table S1). All sequenced reads were aligned to the reference genome Nipponbare (*japonica*) by using BWA version 0.5.9 rc1 [17]. Roughly half of the reads (56.1% in *O. sativa*, 49.8% in *O. rufipogon*) reliably mapped to the reference genome with a Mapping Quality (MAPQ) score of ≥50. On average, we identified 4.2×10^5^ SNPs and 7.4×10^4^ short indels, and 1.5×10^6^ SNPs and 2.3×10^5^ short indels per accession for *japonica* and *indica*, respectively (Table S1). We then delineated 1,042,719 high quality SNPs in 31,036 annotated genes to use in further population genetic analyses (see Text S1 for details).

**Table 1:** Accessions and sampling locations.

No	Accession	Taxon	Sampling Location	ref
1	W593	*rufipogon*	Malaysia	1
2	W1294	*rufipogon*	Philippines	2
3	W1807	*rufipogon*	Sri Lanka	3
4	W2003	*rufipogon*	India	3
5	W1976	*rufipogon*	Indonesia	3
6	W2057	*rufipogon*	Bangladesh	3
7	W0120	*rufipogon*	India	3
8	W630	*rufipogon*	Myammar	4
9	W1866	*rufipogon*	Thailand	3
10	W1965	*rufipogon*	China	2
11	BADARI DHAN (WRC 39)	*Indica*	Nepal	5
12	KALUHEENATL (WRC 41)	*Indica*	Sri Lanka	5
13	KASALATH (WRC 2)	*Indica*	India	5
14	RATUL (WRC 36)	*Indica*	India	5
15	SHONI (WRC 31)	*Indica*	Bangladesh	5
16	SURJAMUKHI (WRC 33)	*Indica*	India	5
17	TUPA121-3 (WRC 32)	*Indica*	Bangladesh	5
18	JENA 035 (WRC 4)	*Indica*	Nepal	5
19	DEEJIAOHUALUO (WRC 98)	*Indica*	China	5
20	HONG CHEUH ZAI (WRC 99)	*Indica*	China	5
21	KEIBOBA (WRC 17)	*Indica*	China	5
22	TAKANARI	*Indica*	Japan	6
23	NIPPONBARE	*Japonica*	Japan	2
24	HITOMEBORE	*Japonica*	Japan	2
25	SASANISHIKI	*Japonica*	Japan	3
26	IWATEKKO	*Japonica*	Japan	2
27	DUNGHAN SHALI	*Japonica*	Hungary	2
28	JAGUARY (WRC 47)	Tropical *japonica*	Brazil	5
29	URASAN1 (WRC 51)	Tropical *japonica*	Japan	5
30	REXMONT (WRC 50)	Tropical *japonica*	United States	5
31	TUPA 729 (WRC 55)	Tropical *japonica*	Bangladesh	5
32	NERICA1	*O. glaberrima* x *japonica*	Africa	7

1: Takano-Kai et al. [60].

2: Rakshit et al. [61].

3: Oryzabase (http://www.shigen.nig.ac.jp/rice/oryzabase/top/top.jsp).

4: Ishikawa et al. [62].

5: NIAS Genebank (http://www.gene.affrc.go.jp/databases-core_collections_wr.php)

6: Imbe et al. [63].

7: AfricaRice (http://www.africarice.org/warda/uplandnerica.asp).

### Genome-wide pattern of SNPs

**Figure 2:**
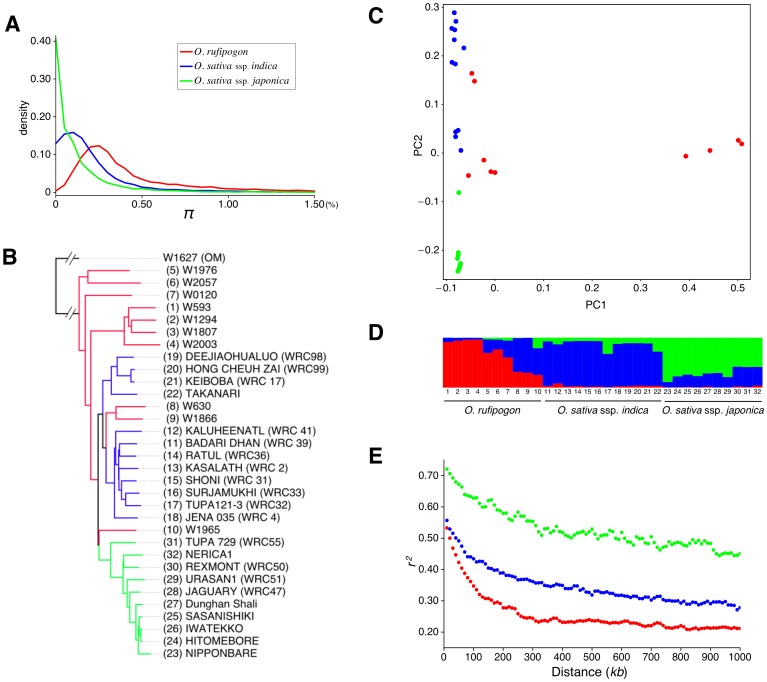
Summary of population genetics analyses. In each figure, red is used for *O. rufipogon*, blue for *indica*, and green for *japonica.* (A) Distribution of π of protein coding genes. The first 1000 synonymous sites from the translation start site were used for each gene to correct for the difference in length in each gene. 13,471 genes with 1,000 sites with reliable SNP data are used. (B) Neighbor-joining tree of all sequenced strains. (C) Population structure estimated by PCA. (D) Population structure estimated by the bayesian clustering program STRUCTURE (*K* = 3). The results of *K* = 2∼6 are shown in Figure S1. (E) Decay of LD against distance. The bin size is 2000 bp (measured until 1000 kb).

In order to identify target regions of selection during the domestication process, it is imperative to obtain an overview of the population structure of each rice species. We first surveyed the level of polymorphism by estimating the average numbers of pairwise nucleotide differences per site, denoted by π (Table 2 and Table S2). Similar to previous studies [8], [18], the level of polymorphism in *O. sativa* was about 60% of that of *O. rufipogon* (Figure 2A). The results for *O. rufipogon*, *indica,* and *japonica* are presented in red, blue, and green, respectively in all figures. Polymorphism is particularly reduced in *japonica* (∼27.5% of *O. rufipogon*). The diversity was reduced in protein-coding sequences (CDS) compared to non-coding sequences, especially in non-synonymous sites, probably because they are under stronger selective constraints. The nucleotide diversity was lower in introns and untranslated regions (UTRs) than in synonymous sites, implying that introns and UTRs might be under stronger selective constraints than synonymous sites (Table S2). This pattern has also been observed in *Drosophila melanogaster* [19]. We then evaluated the population structures by constructing a neighbor-joining (NJ) tree, performing a principal component analysis (PCA), and also by using STRUCTURE [20] (Figure 2B,C,D. See also Figure S1). Consistent with other studies [8], [9], [21], each analysis showed that *O. rufipogon* and the two *O. sativa* subspecies, especially *japonica*, are relatively well differentiated but not completely; there are substantial local variations. We also examined the relationship between the levels of linkage disequilibrium (LD) and the physical distances for *O. rufipogon*, *indica*, and *japonica* populations. LD in *O. rufipogon* decayed as the distance increased, and saturated roughly at 100 kb. The decay was slower in both *indica* and *japonica* compared to *O. rufipogon*, most likely because of the reduced effective population sizes due to the founder (bottleneck) effect in the initial phase of the rice domestication. Another important factor is the selfing rate which directly reduces the efficacy of recombination; *indica* and *japonica* are near selfers with a selfing rate of ∼95% [22], which is much higher than that of *O. rufipogon* (40∼95%, [23]). The decay was especially slow in *japonica* probably because it underwent a more severe bottleneck [21], [24]. The LD pattern suggests that the local patterns of SNPs are shuffled by recombination, and that the correlation between regions that are more than several hundred kb apart is expected to be small. Therefore, in the subsequent analyses, we focused on local patterns of SNPs in the entire genome. The local variation was evaluated by window analyses with different sizes (100 kb, 200 kb and 500 kb). As essentially identical results were obtained for the three sizes of window (not shown), in the following analyses, we use the results obtained with a window size of 500 kb.

**Table 2:** Summary of SNPs and nucleotide diversity.

		All (65,827,877 bp)^1^			CDS (22,536,684 bp)^1^			Non-CDS (43,291,193 bp)^1^		
		SNPs	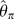 2	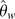 3	SNPs	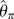 2	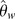 3	SNPs	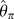 2	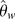 3
All	n = 32	1,042,719	0.32	0.39	256,361	0.23	0.28	786,358	0.36	0.45
*O. rufipogon*	n = 10	855,296	0.40	0.32	210,446	0.29	0.23	644,850	0.46	0.37
*O. sativa*	n = 22	494,458	0.24	0.19	122,504	0.17	0.13	371,954	0.27	0.21
*indica*	n = 12	380,807	0.21	0.14	95,410	0.15	0.10	285,397	0.24	0.16
*japonica*	n = 10	230,093	0.11	0.09	57,316	0.08	0.06	172,777	0.12	0.10

1 Sites from 31,036 genes.

2 Estimator of θ (4*Nµ*) based on the average numbers of pairwise nucleotide differences [64].

3 Watterson’s estimator of θ (4*Nµ*) based on the number of segregating sites [27].

### Detecting selection from local patterns of SNPs

**Figure 3:**
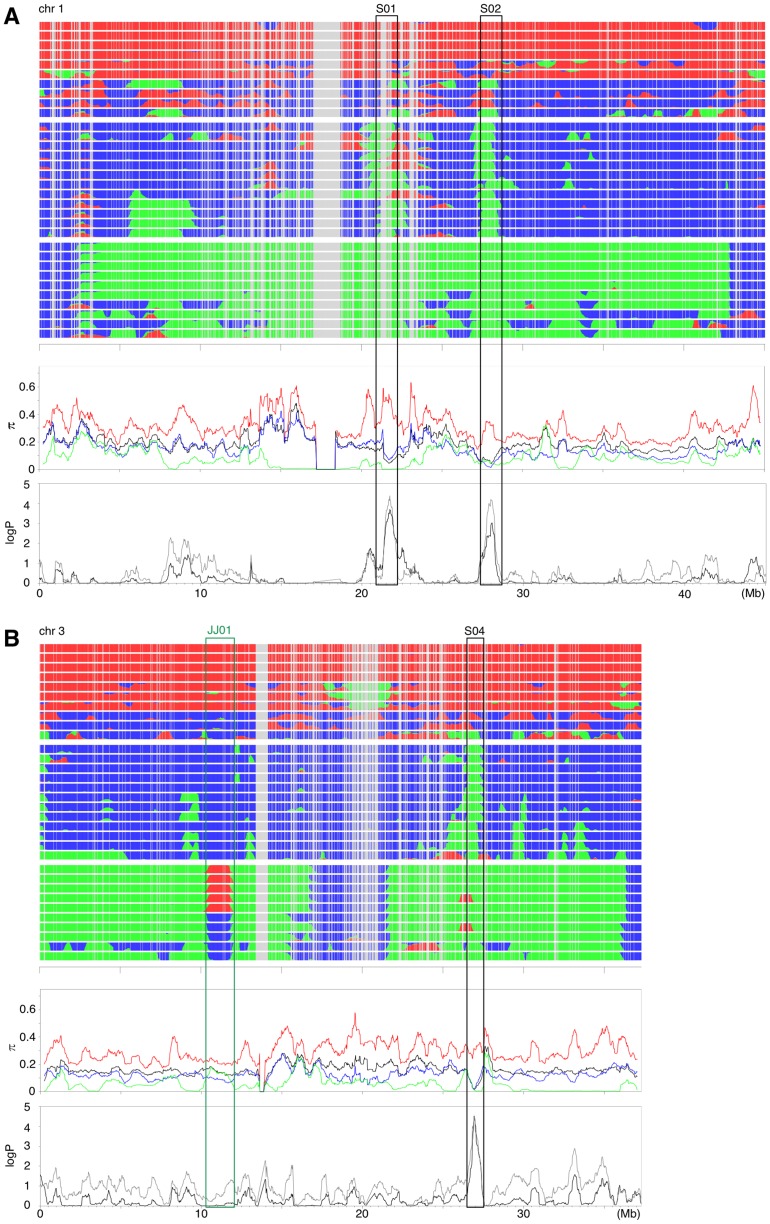
Genome-wide analysis of population structure and π for chromosomes 1 (A) and 3 (B). The upper panel shows the results of STRUCTURE of *K* = 3. Thus, three clusters are assumed, which generally (but not strictly) correspond to *O. rufipogon* (red), *indica* (blue) and *japonica* (green). Unaligned regions (mostly due to gaps) are in gray. The middle panel shows the genome wide distributions of π for each taxa. *O. rufipogon* is in red, *indica* in blue, *japonica,* in green, and *O. sativa* (both *indica* and *japonica* included together) in black. The lower panel shows the statistical scores (logP) of the observed π (black) and θ_w_ (gray) of *O. sativa O. rufipogon*, calculated by coalescent simulation [59]. The top 10 low diversity regions are indicated by black boxes (S01, S02, and S04). The region that shows exceptionally high *F_ST_* between tropical and temperate *japonica* is indicated by a green box (JJ01). Results of the other chromosomes are shown in Figure S3.

**Figure 4:**
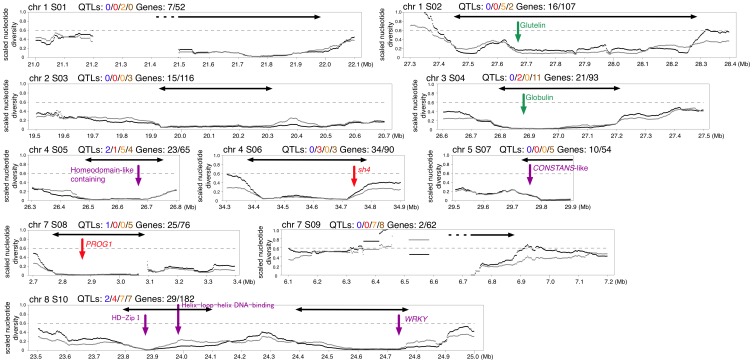
Spatial distribution of the level of polymorphism around the top 10 low diversity regions. The black line shows the scaled π (*O. sativa/O. rufipogon*), and the gray line shows the scaledθ_w_ (*O. sativa/O. rufipogon*). The dotted line indicates the genome-wide average of the scaled π and θ_w_. The positions of two known domesticated genes *sh4* [28], [29] and *PROG1* [30], [31] are indicated by red arrows. The positions of some other interesting candidate domestication genes; seed storage proteins, and a number of transcription factors that contain fixed variants in *O. sativa*, are indicated by green and purple arrows, respectively. The numbers of QTLs related to awn length, shattering, seed dormancy, and quality (in blue, red, orange, and brown, respectively), and the number of annotated genes with fixed variants in *O. sativa* over the total number of annotated genes in each region are indicated. The black arrowed lines indicate the approximate regions of selective sweeps (the selective sweep regions in S01 and S09 are likely to extend into the regions shown by broken lines that could not be analyzed due to low sequencing coverage etc).

Selection during the domestication process should result in a drastic reduction of π in regions of cultivated rice containing the alleles targeted by selection. We searched for such regions with reduced π at synonymous sites in *O. sativa* (π_s_) relative to that in *O. rufipogon* (π_r_). Population bottleneck should also affect the pattern of polymorphism by causing a genome-wide reduction of π_s_. We therefore first estimated the size and duration of bottleneck using a coalescent simulation-based likelihood approach assuming a two-population model that has been commonly used for the analysis of domesticated species (see Text S1 and Figure S2) [5], [25], [26]. Then, assuming these estimated parameters represent the reduction ofπ_s_ at neutral loci (with the least effect of selection), we produced a null distribution ofπ_s_/π_r_ over 500 kb by 100,000 replications of further coalescent simulations. Theπ_s_/π_r_ across each chromosome was then computed with a 500 kb sliding window with steps of 20 kb. We derived scores for each 500 kb window that reflect the statistical significance of the observedπ_s_/π_r_, that is, the log-scaled proportion (logP) of simulation runs with π_s_/π_r_ lower than the observed value. In Figure 3, the spatial distributions of π_r_ and π_s,_ and the statistical scores are plotted along the local clustering patterns obtained by the linkage model of STRUCTURE (results for chromosomes 1 and 3 are shown here, see Figure S3 for the remaining chromosomes). Overall, π_s_ is lower than π_r_. However, several regions with reduced genetic variation in *O. sativa*, as exemplified by lowπ_s_/π_r_ and high statistical scores, were observed. These regions generally corresponded to regions with less differentiation between *indica* and *japonica* in the local STRUCTURE pattern, where all *indica* and *japonica* strains are represented by the same color (either blue or green) (Figure 3). We also evaluated the statistical significance by using another measure of polymorphism level based on the number of segregating sites (θ_W_, [27]), and obtained almost identical results (see Figure 3 and Figure S3). Each 500 kb region was ordered according to the statistical scores based on the π_s_/π_r_ and θ_s_/θ_r_ ratios. The ten regions containing 500 kb regions that were within the top 15 by both measures are indicated by black boxes (S01 to S10; S10 might contain two different regions) in Figure 3 and Figure S3. We specifically focused on these regions that most probably were targeted by selection during the domestication process. Fine-scale distributions of the genetic variation across these ten regions of extremely low variation are shown in Figure 4. We found that two of these 10 regions contain “known” domestication genes; *sh4* [28], [29] was present in a region on chromosome 4 (Figure 4, S06) and *PROG1* [30], [31] in a region on chromosome 7 (Figure 4, S08). These 10 regions overlap but are not identical with selective sweep regions reported by other recent rice resequencing studies. Eight out of the 10 (apart from S01 and S03) overlap with the regions reported by [9], which are based on the ratio ofπ_r_/π_s_. Another eight out of 10 (apart from S02 and S09) overlap with regions reported by [7] where the *F_ST_* between *indica* and *japonica* were significantly smaller than both the *F_ST_* between *O. rufipogon* and *indica* and between *O. rufipogon* and *japonica.* Six out of 10 (S02, S04, S05, S06, S08, and S10) overlap with those reported by [8], which are based on the ratio of diversity in *indica* to the diversity of *O. nivara* (π_i_/π_nivara_).

### Detecting selection involved in the diversification process of rice

Although the origin of cultivated rice is still under debate, the two subspecies *indica* and *japonica*, and also the two different groups of *japonica*, temperate *japonica* and tropical *japonica* should have experienced different directional selection. We can also identify such selection using similar methods. First, we looked for regions that exhibit reduced genetic variation within each subspecies by applying the same analysis as described above to the *O. rufipogon-indica* and *O. rufipogon-japonica* pairs separately. In practice, we inferred the demographic parameters using the two-population model, and the statistical significances (based on the π and θ_W_ ratios) were evaluated considering the inferred demography.

**Figure 5:**
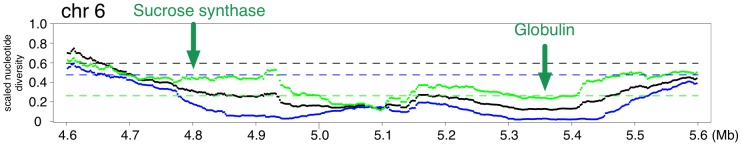
Spatial distribution of the level of polymorphism around a typical low diversity region in *O. sativa* ssp. *indica*. The statistical scores of the scaled π of *indica/O. rufipogon*. The dotted horizontal lines indicate the genome-wide average values of π. *Indica* is shown in blue, *japonica* in green, and *O. sativa* (*indica* and *japonica* pooled) in black. Results of the entire genome are shown in Figure S4.

The obtained scores for each 500 kb region across each chromosome are shown in Figures S4 and S5. For the *O. rufipogon-indica* comparison, we found that the distributions of π_i_/π_r_ and θ_i_/θ_r_ were very similar to those of the *O. rufipogon-O. sativa* pair, indicating that the majority of the genetic variation in *O. sativa* can be explained by the variation in *indica*. Accordingly, the detected regions of reduced diversity for the *O.rufipogon-indica* pair are very similar to those for the *O. rufipogon-O. sativa* pair. We found 11 low diversity regions that were within the top 15 based on bothπ_i_/π_r_ and θ_i_/θ_r_. 8 of the 11 regions overlapped with the 10 low diversity regions based on the *O. rufipogon-O. sativa* comparison. Two of the remaining three low diversity regions did show reduced diversity in *O. sativa* but were not in the top 10 regions. Only one region showed clear reduction of diversity specifically in *indica* and not in *japonica* (Figure 5, see also the blue-boxed region, I01, in Figure S3F). It is most likely that this region has undergone selection specific to *indica*.

**Figure 6:**
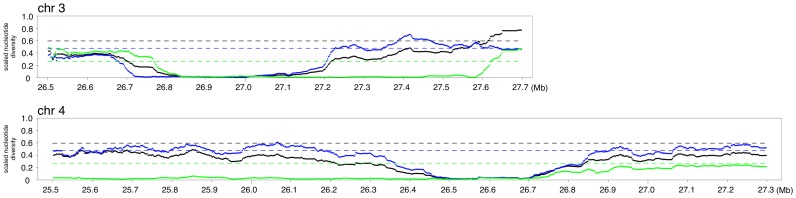
Spatial distribution of the level of polymorphism around two typical low diversity regions in *O. sativa* ssp. *japonica*. The statistical scores of the scaled π of *japonica/O. rufipogon*. The dotted horizontal lines indicate the genome-wide average values of π. *Indica* is shown in blue, *japonica* in green, and *O. sativa* (*indica* and *japonica* pooled together) in black. Results of the entire genome are shown in Figure S5.

By contrast, we obtained quite a different picture for the *O. rufipogon-japonica* comparison. In *japonica*, the genetic diversity was generally low throughout the genome, and a number of large regions exhibited almost no genetic variation. Two examples are shown in Figure 6; a typical pattern is that a region of reduced polymorphism in *japonica* is much wider than that detected by the *O. rufipogon-O. sativa* comparison. Therefore, the data of *japonica* is not very suitable to narrow down the target of selection.

**Figure 7:**
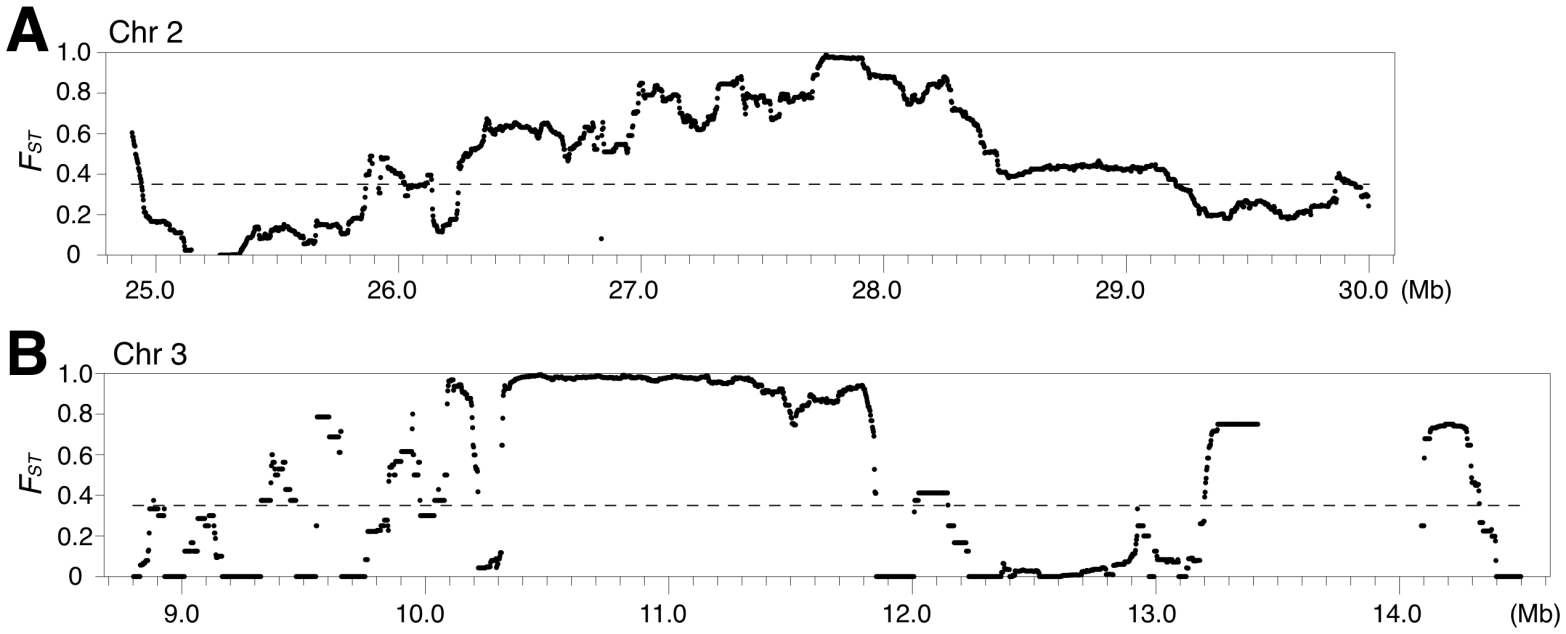
Highest *F_ST_* regions. (A) Between *japonica* and *indica*. (B) Between temperate *japonica* and tropical *japonica.*

We also searched for regions that are highly differentiated between *indica* and *japonica* by computing the *F_ST_* between the two subspecies for the entire genome. We found one region on chromosome 2 that showed especially high *F_ST_* (Figure 7A, see also the green-boxed region, IJ01, in Figure S3B). We also computed the *F_ST_* between the temperate and tropical *japonica* accessions. One region on chromosome 3 in particular showed a strikingly high degree of differentiation (Figure 7B, see also the green-boxed region, JJ01, in Figure 3B or Figure S3C). This can also be observed by the STRUCTURE analysis (Figure 3B). These two regions have most probably been under strong directional selection and are likely to be responsible for key phenotypic differences between *indica* and *japonica*, or temperate and tropical *japonica,* respectively.

### Detecting candidate target genes of selection from QTL information

In the previous sections, we overviewed the genome-wide patterns of SNPs, from which we identified local regions that were likely targeted by domestication selection. One kind of signature of selection is the local reduction of the level of SNPs (Figures 3 and 4). This is most probably due to strong selection on a beneficial allele of a certain gene sweeping out other genetic variants in the nearby linked regions, as in *sh4* in S06 and *PROG1* in S08 (Figure 4). The identification of such domestication genes is of huge agronomic importance and is the ultimate goal of many domestication-related studies. However, as shown here and also in other rice resequencing studies [7], [8], [9], the low diversity regions in *O. sativa* are large and contain a large number of genes (tens or even over a hundred), making it difficult to determine the exact targets. As it is unrealistic to experimentally test every single gene, it is desirable to further narrow down the candidates based on *in silico* approaches. To this end, He et al. [7] reported genes within low diversity regions that have at least one nonsynonymous substitution distinguishing the cultivated species from the wild species. However, this approach is potentially misleading and also restrictive because the target of selection could be indels instead of point mutations, various loss-of-function mutations, or mutations in regulatory regions that affect the expression of the gene, as observed in many previously reported domestication alleles [3], [13], [14].

Here, we have instead used a more inclusive approach and have provided various information on the low diversity regions that should facilitate further in-depth investigations. In particular, the excellent resource of QTLs in rice should be useful in narrowing down the target genes. The GRAMENE database [32] contains several kinds of QTLs whose rough locations have been determined by QTL mapping [15]. We first compiled a list of QTLs in GRAMENE that map close to each of the 10 selective sweep regions. As mentioned in the Introduction (see also Figure 1), the domestication process should have targeted a number of quantitative traits. It is then reasonable to assume that the selective sweep regions should be strongly linked to some of the QTLs that were targeted by domestication selection, such as shattering, seed dormancy, awn length, and grain quality. If so, they should contain genes with functions that are related to these QTLs. We have listed all QTLs that overlap with each target region of selection (Dataset S1). We have particularly highlighted QTLs related to shattering, awn length, seed dormancy, and grain quality that map close to the low diversity regions in Figure 4. In addition, we have listed all genes within each low diversity regions, together with the expression evidence of each genes in 7 different tissues based on the RNA-seq analysis of [16] (Dataset S2). We have also listed specifically those that have variants including point mutations and indels in coding regions or upstream regions that are fixed in domesticated rice (Dataset S3). We note that our data do not cover all point mutations and indels in these candidate regions because there are regions where short-reads are difficult to map. Although this problem applies to any study based on next-generation short-read sequencing, the proportion of such regions is smaller compared with previous studies of rice [7], [8], [9] because of the much higher coverage achieved in this study. We also acknowledge that the resolution of QTL mapping is often low and the “genomic location” of a mapped QTL can sometimes span a few megabases and also be imprecise. This makes it difficult to confidently assign any given QTL to a selective sweep region or assign a gene to every QTL. We have here chosen to be inclusive rather than restrictive, and our list of QTLs should contain several QTLs that are not associated with the selective sweep regions. The list of QTLs should thus be considered as an additional source of information that will help narrow down the candidate target genes. Furthermore, there would be other traits that one could potentially associate with the initial domestication or subsequent diversification processes. Our aim here is not to be restrictive or to try to single out one particular QTL or gene, but to provide enough useful information that should allow researchers to generate various hypotheses which can then be experimentally tested.

## Discussion

Understanding the genetic basis of important phenotypes is one of the major goals of molecular biology, especially in cultivated plants because the identification of genes underlying agronomic traits can directly contribute to the further improvement of the yield and quality [1]. Although QTL mapping, and more recently population genetic approaches, are commonly used to search for genes (or genomic regions) responsible for phenotypic differences, the actual identification of such genes remains a huge challenge [3]. *O. sativa* and its wild progenitor *O. rufipogon* are clearly different in several traits related to the yield and quality of rice, and selection should have played a crucial role in genes related to such traits during the domestication process of rice. A genome-wide population genetic survey of selection should identify large regions containing the target genes, and the QTL information should serve as a powerful guide to narrow down on them. Below we discuss a few interesting examples.

QTLs related to shattering, awn length, and seed dormancy all mapped to the selective sweep regions S05 and S10 (Figure 4). This suggests that mutations in genes controlling these traits within these regions were likely to be targeted by selection. Transcription factors would be strong candidates as they can affect multiple developmental traits including seed shattering and dormancy [33], [34], [35], and many genes identified as controlling domestication traits are transcription factors [3]. We found that a homeodomain-like containing gene in S05 has a nucleotide variation in the 5’ upstream region that is fixed in *O. sativa*. We also found 3 transcription factor genes in S10 which contain sequence variants fixed in *O. sativa* – a homeoldomain leucine zipper (HD-Zip) I gene with a deletion of a single amino acid (3 nucleotides), and a helix-loop-helix DNA binding gene and a WRKY gene with nonsynonymous substitutions in protein-coding regions (Figure 4 and Dataset S3). All 4 genes have expression evidence in *O. sativa* (Dataset S2). In addition, it is thought that some HD-Zip I and *WRKY* genes are involved in the abscisic acid (ABA) signaling pathway and control seed dormancy and germination [34], [36], [37]. It is therefore tempting to speculate that a mutation in one of these transcription factors was selected for due to its pleiotropic effect on multiple domestication traits.

Grain quality, which includes eating, cooking, nutritional, and milling quality, clearly differentiates wild and cultivated rice and might well have been targets throughout human cultivation. In particular, seed storage protein genes would be strong candidates as they account for a significant portion of the total protein content of seeds, and strongly affect the nutritional quality of rice [38]. In addition, the major seed storage proteins, globulin, prolamin, and glutelin, are highly expressed in seeds [39], [40]. We found that S04 has 11 quality-related QTLs, and also contains a Globulin-1 gene which has two nucleotide variations in the upstream promoter region that are fixed in the *O. sativa* accessions. Interestingly, this gene was reported to be up-regulated in a high milling yield cultivar Cypress compared to a low milling yield cultivar LaGrue [41]. We also noticed that several quality-related QTLs map close to the region that showed reduction of diversity specific to *indica* (Figure 5). This region contains a globulin-like gene, and also a sucrose synthase 2 gene. Sucrose synthase catalyzes the first step in the conversion of sucrose to starch and may well affect various quality traits such as amylose content, gel consistency, or gelatinization temperature [42], [43]. Genes involved in starch biosynthesis are thought to have been under strong selection during domestication in both maize and rice [8], [44]. It has also been reported that sucrose synthase activity affects rice grain yield [45].

Another interesting candidate is the *CONSTANS*-like gene in S07, which has a fixed deletion in the 5’ upstream region and a fixed nonsynonymous substitution in the protein-coding region (Figure 4). *CONSTANS* genes are transcription factors that have an important role in the controlling of flowering time, which should have been a major determinant for the adaptation of cultivated rice to different environments [46]. Two recent studies suggested that *Hd1*, ortholog of the *Arabidopsis CONSTANS* gene, might have been targeted by human selection during the domestication process [47], [48]. Interestingly, a QTL that affects days to heading and days to maturity (*i.e.* flowering time) is mapped to this region (Dataset S1).

We were also able to identify some interesting candidates in the region highly differentiated between *indica* and *japonica*, and in the region highly differentiated between temperate and tropical *japonica* (Figure 7). *Indica* is a lowland rice that is usually grown submerged throughout tropical Asia, whereas *japonica* is usually an upland rice cultivated in dry fields. Upland rice has thus developed drought-resistant traits and often has a deeper root system in response to water deficit conditions [49]. We noticed that the region on chromosome 2 (Figure 7A) contained several QTLs related to different aspects of root. In addition, this region contained an aquaporin gene *OsPIP1;1* (*OsPIP1a*). Aquaporins play an essential role in water uptake and water movement. *OsPIP1;1* is expressed in root, and its expression is regulated in response to water stress or drought treatments [50], [51]. Although *OsPIP1;1* did not exhibit any difference in expression between the *indica* and *japonica* accessions in these particular studies [50], [51], it would be interesting to examine whether this gene has different functions in *indica* and *japonica*, and whether it might have contributed to the different adaptation of these two subspecies.

Tropical *japonica* is cultivated in Southeast Asia such as Indonesia and the Philippines, whereas temperate *japonica* is cultivated in temperate East Asia and regions of higher altitudes in South Asia and Southeast Asia. Temperate *japonica* is thought to be derived from tropical *japonica*. This process most probably involved artificial selection for traits such as cold tolerance. We found that the highly differentiated region on chromosome 3 (Figures 3B and 7B) contains a gene *OsCIPK03*, also known as *OsCK1*, whose expression was shown to be induced by diverse signals including cold [52]. Furthermore, transgenic plants overexpressing this gene showed improved tolerance to cold by being able to accumulate higher contents of proline and soluble sugar during cold stress compared to wild type plants [53]. Other candidate targets within this region are a number of *MYB* family transcription factor genes, a large family of transcription factor genes that are known to be involved in response to various stresses including cold [54]. A QTL for low-temperature vigor has also been mapped to this region [55], although this QTL was not present in GRAMENE.

These candidates are obviously not conclusive and other genes could well have been the targets of selection. Nevertheless, they should be more biologically relevant and meaningful than those that are only based on sequence information. In addition, the list of QTLs and genes within each region that we provide here should allow researchers to investigate other possibilities.

Bottom-up population genomic analysis with genome resequencing is likely to become more and more of a common approach to search for genes responsible for phenotypic differences, especially in model organisms. Indeed, a few other studies have recently reported the resequencing of different accessions of wild and cultivated rice [7], [8], [9]. We also sequenced each accession to a high coverage as in [8] rather than pooling many accessions together as in [7], and our sequence data should be a useful resource for further rice population studies. Furthermore, we have here shown that by combining the population genomic information with other valuable information such as QTLs, we can gain a lot more insight and make a more informed decision on candidate domestication genes to further investigate. Although we mainly focused here on differences between cultivated and wild rice, we were also able to identify strong candidate targets of artificial selection in highly divergent regions between *indica* and *japonica*, and between tropical and temperate *japonica*, which was not done in the other studies [7], [8], [9]. Our approach should therefore be applicable to other more specifically designed resequencing studies such as to identify genes related to the local adaptation of certain landraces.

## Materials and Methods

### Genome sequencing and SNP calling

Sequencing libraries were constructed according to the manufacturer’s instruction (Illumina). Paired-end short reads (75-bp) were generated using the Illumina Genome Analyser IIx systems. Low quality reads that contain contiguous undetermined nucleotides or a long array of a single kind of nucleotide were removed. The obtained paired-end short reads were then mapped to the *O. sativa cv.* Nipponbare reference genome (IRGSP build 5, masked with MIPS repeat data) using the short-read alignment program BWA version 0.5.9 rc1 [17]. Reliably mapped reads (Map quality ≥50) were used in subsequent analyses. SNP detection was conducted using Samtools ver. 0.0.12a without BAQ algorithm [56]. To avoid false positive and false negative errors, we screened for SNPs with the SNP quality score ≥100 and depth ≥3. We also excluded SNPs with depth ≥100 because such SNPs are likely located in repetitive regions or TEs. The raw sequence data is available at NCBI under the accession number PRJNA222757.

### Analyses of genome-wide SNP patterns

We delineated high quality SNPs within gene regions to minimize the risk of comparing paralogous sequences across multiple individuals caused by repetitive regions including transposable elements (TEs) (see Text S1 for details). These SNPs were used to calculate π and θ_w_. Once candidate target regions of selection with reduced nucleotide diversity were identified, π and θ_w_ of these regions were calculated using all sites including those outside gene regions in order to obtain a finer picture by increasing the sample size. The SNPs within the gene regions were also used for the following analyses. An NJ tree was constructed using the PHYLIP package based on the pairwise p-distance calculated using all sites within the gene regions. Sites containing missing data among the 32 accessions or the outgroup accession were excluded. *O. meridionalis* was included as an outgroup which we had also sequenced at a low coverage. The model-based program STRUCTURE 2.3 [20] was used to evaluate the genetic structure among the 32 accessions. For this analysis, a random set of SNPs was used to represent the genome (roughly one SNP every 20 kb). First, the correlated allele frequency model and admixture model with no linkage was used. The posterior probability of *K* from 2 to 9 under the no linkage model was calculated to infer the number of clusters *K.* Five independent runs yielded nearly consistent results for each *K* (Figure S1). The highest posterior probability was obtained when *K* = 3. The three clusters were more or less consistent with the *O. rufipogon*, *indica,* and *japonica* populations. Next, the linkage model with *K* = 3 assuming constant recombination rate across the entire genome was used to infer the patterns of genetic structures across chromosomal regions. Principal component analysis (PCA) was performed using a random set of SNPs (roughly one SNP every 50 kb). The eigenvectors were calculated by the procmp function of the R statistical package [57], [58]. The relationship between the levels of linkage disequilibrium (LD) and the physical distances for wild, *indica* and *japonica* populations was evaluated by calculating the *r^2^* statistic using all SNPs. 10 out of 12 individuals from the *indica* population were randomly selected for each pair of SNPs so that we can compare the results of the three populations with the same sample size.

### QTL information

A list of QTLs was downloaded from the GRAMENE database (http://www.gramene.org). The 11,624 QTLs are classified into 9 large categories, which are further divided into 332 traits [15]. The midpoint of the start and end positions of each QTL was treated as the position of the QTL. QTLs whose start and end positions were more than 5 Mb apart were removed, which resulted in 6,862 QTLs remaining. Of these QTLs, 1,017 mapped to the low diversity regions (+/−2Mb), and these are listed in Dataset S1.

### Screening of genes with fixed mutations

We screened all gene regions in each low diversity region for replacement or frameshift mutations that are fixed in cultivated rice. We also searched for mutations in the 200 bp upstream or 100 bp downstream regions of annotated transcription start sites. All sites containing such mutations were reported if *F_ST_* ≥0.7 and if the variant is fixed in cultivated rice. Only sites where ≥6 accessions in *O. rufipogon*, and ≥12 in *O. sativa* (≥6 in *indica* in the case of the low diversity region specific to *indica*) had determined nucleotides (not ‘N’) were considered.

## Supporting Information

Figure S1 **Results of STRUCTURE with** ***K*** **= 2∼6. Red, blue, and green roughly correspond to** ***O. rufipogon*** **,** ***indica*** **and** ***japonica*** **, respectively.**
(TIFF)

Figure S2 **Estimation of demographic parameters.** (A) The demographic model used in this study. (B) The log likelihood distribution for *N*_0_. The maximum likelihood estimate is indicated by a vertical line at *N*_0_ = 180×10^3^. (C-D) The two-dimensional distribution of log likelihood for *T*_1_ and *N*_1_ for the *O. rufipogon – O. sativa* (C), *O. rufipogon – indica* (D), *O. rufipogon – japonica* pairs (E). The maximum likelihood estimate is indicated by a red box in each panel.
(TIFF)

Figure S3 **Genome-wide analysis of population structure and π for each chromosome.** For each chromosome, the upper panel shows the results of STRUCTURE. Unaligned regions (mostly due to gaps) are in gray. The middle panel shows the genome wide distributions ofπ for each taxa. *O. rufipogon* is in red, *indica* in blue, *japonica* in green, and *O. sativa* (both *indica* and *japonica* included together) in black. The lower panel shows the statistical scores (logP) of the observed π (black) and θ_w_ (gray) of *O. sativa/O. rufipogon*, calculated by coalescent simulation [59]. The top 10 low diversity regions are indicated by black boxes (S1 to S10). The region that shows reduction of diversity specifically in *indica* is indicated by a blue box (I01). The regions that show exceptionally high *F_ST_* between *indica* and *japonica*, and between tropical and temperate *japonica* are indicated by green boxes (IJ01 and JJ01, respectively).
(ZIP)

Figure S4 **Statistical scores of the observed π and θ_w_ of** ***indica/O. rufipogon*** **.** The black line represents π, and the gray line represents θ_w_, both calculated by coalescent simulation [59].
(TIFF)

Figure S5 **Statistical scores of the observed π and θ_w_ of** ***japonica/O. rufipogon*** **.** The black line represents the score based on π, and the gray line represents that forθ_w_.
(TIFF)

Table S1 **Summary of sequencing.**
(DOCX)

Table S2 **Summary of SNPs and nucleotide diversity.**
(DOCX)

Text S1 **Supplementary methods.**
(DOCX)

Dataset S1 **QTLs mapped to each low diversity region and high** ***F_ST_*** **region.**
(XLS)

Dataset S2 **Genes mapped to each low diversity region and high** ***F_ST_*** **region.**
(XLS)

Dataset S3 **Sites with variants fixed in cultivated rice and** ***F_ST_*** **≥ 0.7 in gene regions of each low diversity region.**
(XLS)
