# Supplementary figures and images for: QTL Map Meets Population Genomics: An Application to Rice
Source: PLoS One. 2013 Dec 23;8(12):e83720. doi: 10.1371/journal.pone.0083720 (PMC3871663; doi:10.1371/journal.pone.0083720)

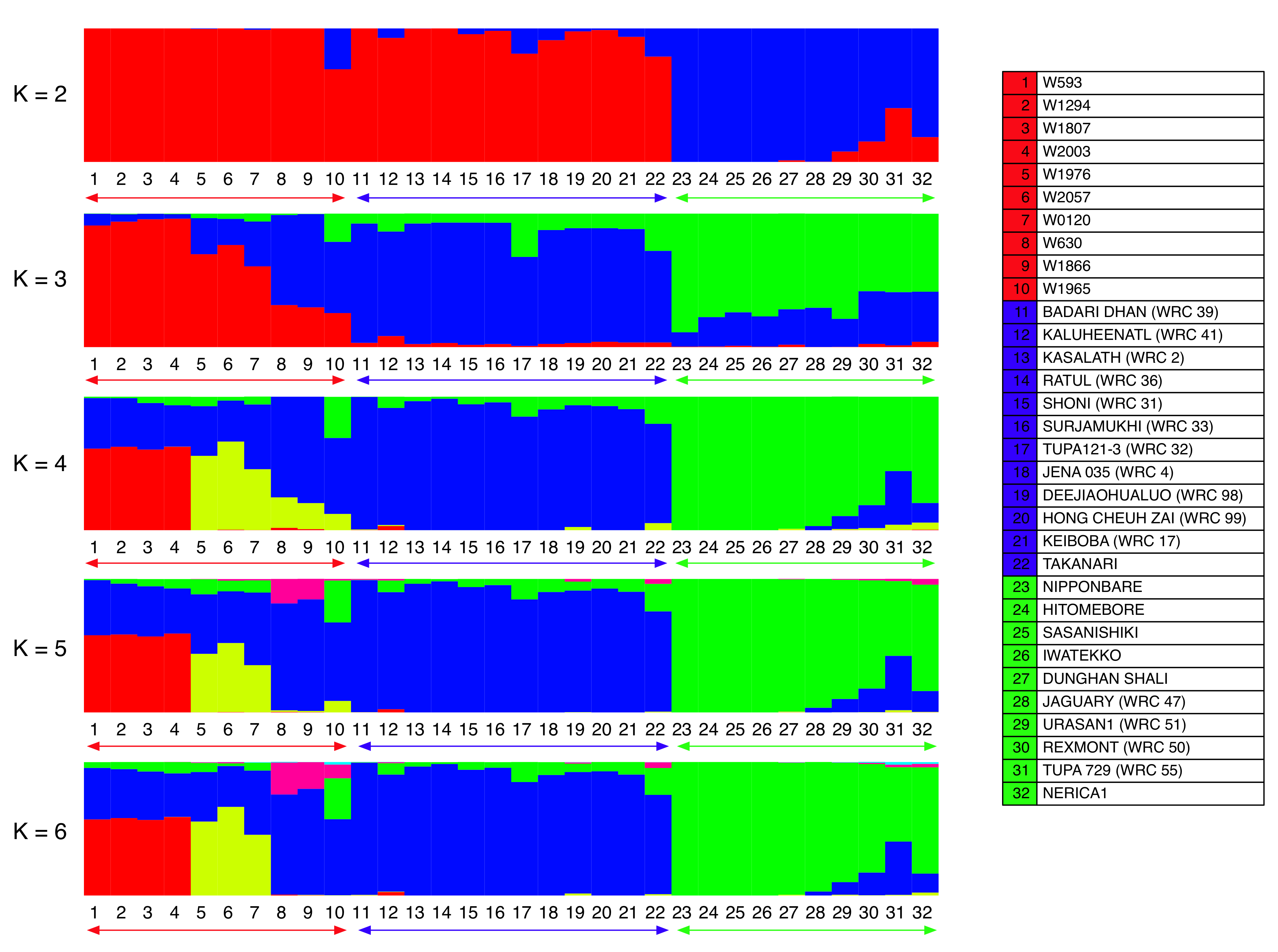

Supplement: Figure S1 — Results of STRUCTURE with K = 2∼6. Red, blue, and green roughly correspond to O. rufipogon , indica and japonica , respectively. (TIFF) [file pone.0083720.s001.tiff]

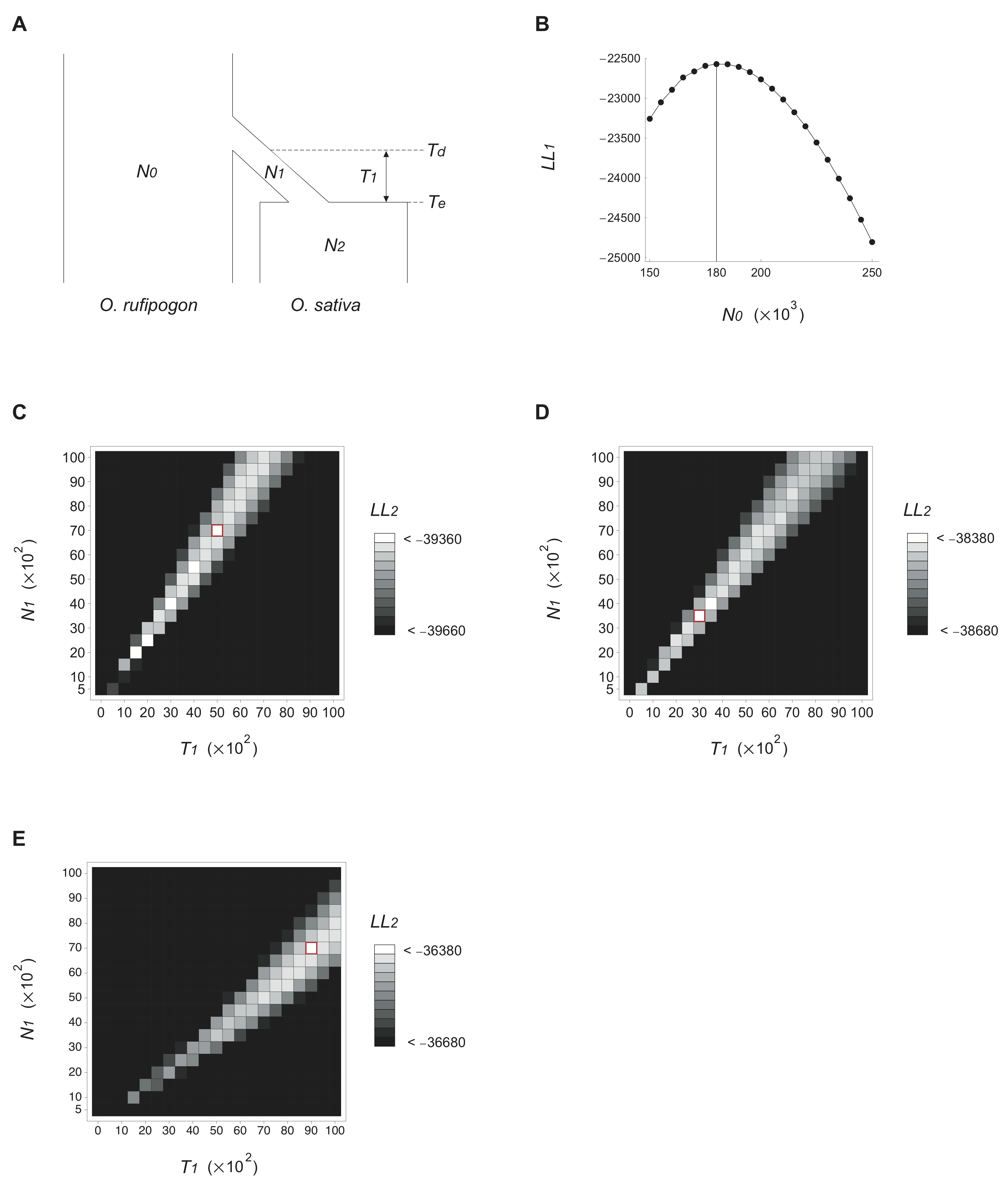

Supplement: Figure S2 — Estimation of demographic parameters. (A) The demographic model used in this study. (B) The log likelihood distribution for N 0. The maximum likelihood estimate is indicated by a vertical line at N 0 = 180×103. (C-D) The two-dimensional distribution of log likelihood for T 1 and N 1 for the O. rufipogon – O. sativa (C), O. rufipogon – indica (D), O. rufipogon – japonica pairs (E). The maximum likelihood estimate is indicated by a red box in each panel. (TIFF) [file pone.0083720.s002.tiff]

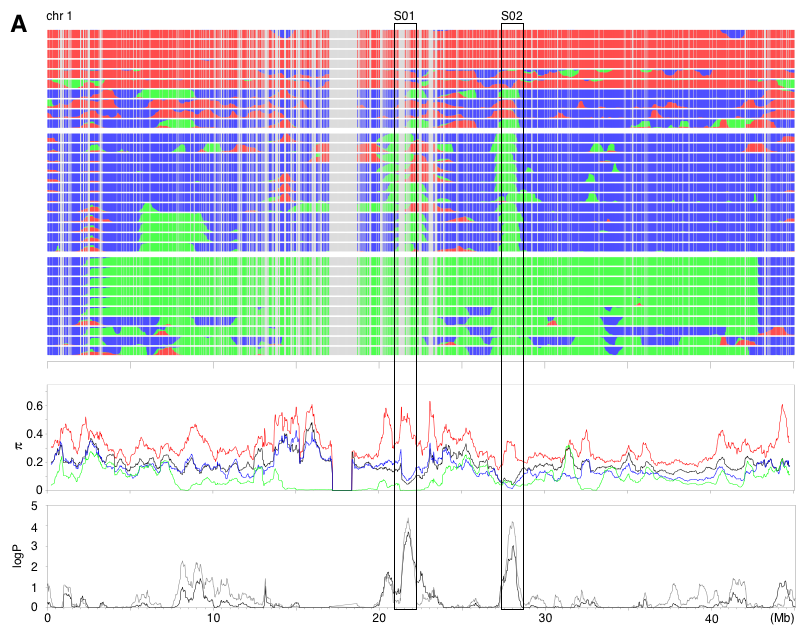

Supplement: Figure S3 — Genome-wide analysis of population structure and π for each chromosome. For each chromosome, the upper panel shows the results of STRUCTURE. Unaligned regions (mostly due to gaps) are in gray. The middle panel shows the genome wide distributions ofπ for each taxa. O. rufipogon is in red, indica in blue, japonica in green, and O. sativa (both indica and japonica included together) in black. The lower panel shows the statistical scores (logP) of the observed π (black) and θw (gray) of O. sativa/O. rufipogon, calculated by coalescent simulation [59]. The top 10 low diversity regions are indicated by black boxes (S1 to S10). The region that shows reduction of diversity specifically in indica is indicated by a blue box (I01). The regions that show exceptionally high FST between indica and japonica, and between tropical and temperate japonica are indicated by green boxes (IJ01 and JJ01, respectively). (ZIP) [file pone.0083720.s003.zip › FigureS3/chr01.png]

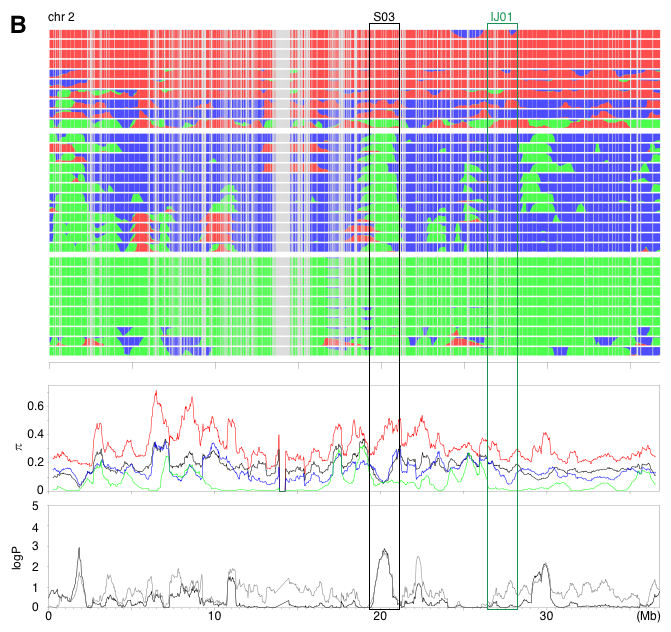

Supplement: Figure S3 — Genome-wide analysis of population structure and π for each chromosome. For each chromosome, the upper panel shows the results of STRUCTURE. Unaligned regions (mostly due to gaps) are in gray. The middle panel shows the genome wide distributions ofπ for each taxa. O. rufipogon is in red, indica in blue, japonica in green, and O. sativa (both indica and japonica included together) in black. The lower panel shows the statistical scores (logP) of the observed π (black) and θw (gray) of O. sativa/O. rufipogon, calculated by coalescent simulation [59]. The top 10 low diversity regions are indicated by black boxes (S1 to S10). The region that shows reduction of diversity specifically in indica is indicated by a blue box (I01). The regions that show exceptionally high FST between indica and japonica, and between tropical and temperate japonica are indicated by green boxes (IJ01 and JJ01, respectively). (ZIP) [file pone.0083720.s003.zip › FigureS3/chr02.png]

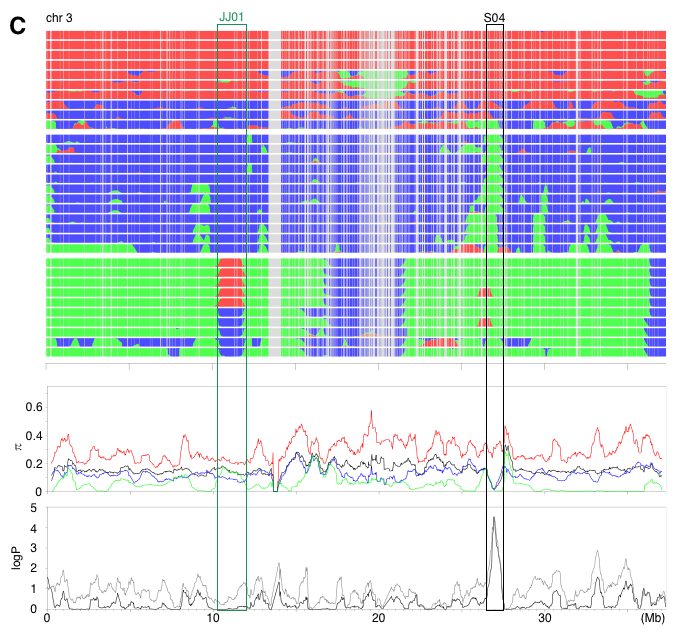

Supplement: Figure S3 — Genome-wide analysis of population structure and π for each chromosome. For each chromosome, the upper panel shows the results of STRUCTURE. Unaligned regions (mostly due to gaps) are in gray. The middle panel shows the genome wide distributions ofπ for each taxa. O. rufipogon is in red, indica in blue, japonica in green, and O. sativa (both indica and japonica included together) in black. The lower panel shows the statistical scores (logP) of the observed π (black) and θw (gray) of O. sativa/O. rufipogon, calculated by coalescent simulation [59]. The top 10 low diversity regions are indicated by black boxes (S1 to S10). The region that shows reduction of diversity specifically in indica is indicated by a blue box (I01). The regions that show exceptionally high FST between indica and japonica, and between tropical and temperate japonica are indicated by green boxes (IJ01 and JJ01, respectively). (ZIP) [file pone.0083720.s003.zip › FigureS3/chr03.png]

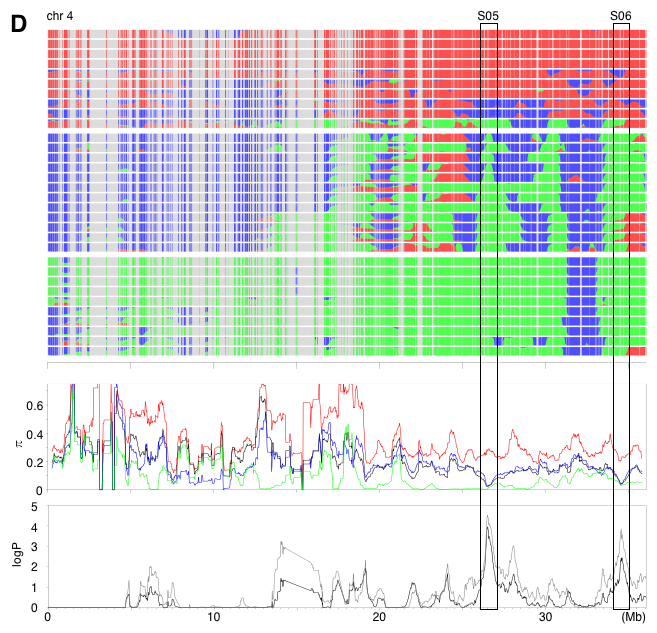

Supplement: Figure S3 — Genome-wide analysis of population structure and π for each chromosome. For each chromosome, the upper panel shows the results of STRUCTURE. Unaligned regions (mostly due to gaps) are in gray. The middle panel shows the genome wide distributions ofπ for each taxa. O. rufipogon is in red, indica in blue, japonica in green, and O. sativa (both indica and japonica included together) in black. The lower panel shows the statistical scores (logP) of the observed π (black) and θw (gray) of O. sativa/O. rufipogon, calculated by coalescent simulation [59]. The top 10 low diversity regions are indicated by black boxes (S1 to S10). The region that shows reduction of diversity specifically in indica is indicated by a blue box (I01). The regions that show exceptionally high FST between indica and japonica, and between tropical and temperate japonica are indicated by green boxes (IJ01 and JJ01, respectively). (ZIP) [file pone.0083720.s003.zip › FigureS3/chr04.png]

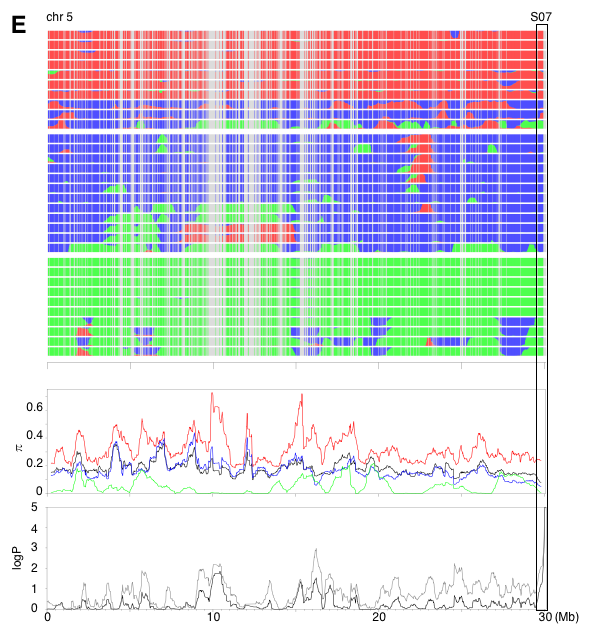

Supplement: Figure S3 — Genome-wide analysis of population structure and π for each chromosome. For each chromosome, the upper panel shows the results of STRUCTURE. Unaligned regions (mostly due to gaps) are in gray. The middle panel shows the genome wide distributions ofπ for each taxa. O. rufipogon is in red, indica in blue, japonica in green, and O. sativa (both indica and japonica included together) in black. The lower panel shows the statistical scores (logP) of the observed π (black) and θw (gray) of O. sativa/O. rufipogon, calculated by coalescent simulation [59]. The top 10 low diversity regions are indicated by black boxes (S1 to S10). The region that shows reduction of diversity specifically in indica is indicated by a blue box (I01). The regions that show exceptionally high FST between indica and japonica, and between tropical and temperate japonica are indicated by green boxes (IJ01 and JJ01, respectively). (ZIP) [file pone.0083720.s003.zip › FigureS3/chr05.png]

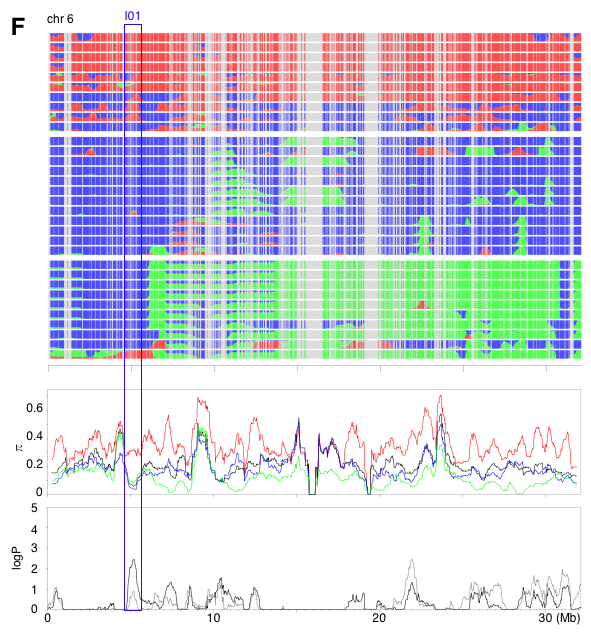

Supplement: Figure S3 — Genome-wide analysis of population structure and π for each chromosome. For each chromosome, the upper panel shows the results of STRUCTURE. Unaligned regions (mostly due to gaps) are in gray. The middle panel shows the genome wide distributions ofπ for each taxa. O. rufipogon is in red, indica in blue, japonica in green, and O. sativa (both indica and japonica included together) in black. The lower panel shows the statistical scores (logP) of the observed π (black) and θw (gray) of O. sativa/O. rufipogon, calculated by coalescent simulation [59]. The top 10 low diversity regions are indicated by black boxes (S1 to S10). The region that shows reduction of diversity specifically in indica is indicated by a blue box (I01). The regions that show exceptionally high FST between indica and japonica, and between tropical and temperate japonica are indicated by green boxes (IJ01 and JJ01, respectively). (ZIP) [file pone.0083720.s003.zip › FigureS3/chr06.png]

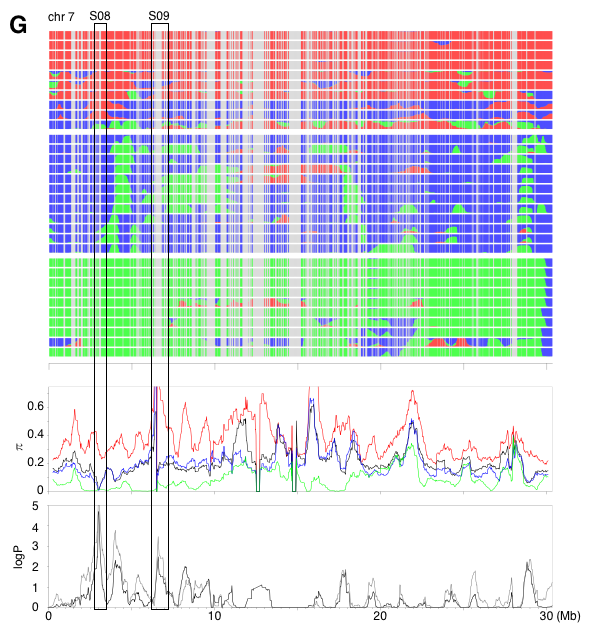

Supplement: Figure S3 — Genome-wide analysis of population structure and π for each chromosome. For each chromosome, the upper panel shows the results of STRUCTURE. Unaligned regions (mostly due to gaps) are in gray. The middle panel shows the genome wide distributions ofπ for each taxa. O. rufipogon is in red, indica in blue, japonica in green, and O. sativa (both indica and japonica included together) in black. The lower panel shows the statistical scores (logP) of the observed π (black) and θw (gray) of O. sativa/O. rufipogon, calculated by coalescent simulation [59]. The top 10 low diversity regions are indicated by black boxes (S1 to S10). The region that shows reduction of diversity specifically in indica is indicated by a blue box (I01). The regions that show exceptionally high FST between indica and japonica, and between tropical and temperate japonica are indicated by green boxes (IJ01 and JJ01, respectively). (ZIP) [file pone.0083720.s003.zip › FigureS3/chr07.png]

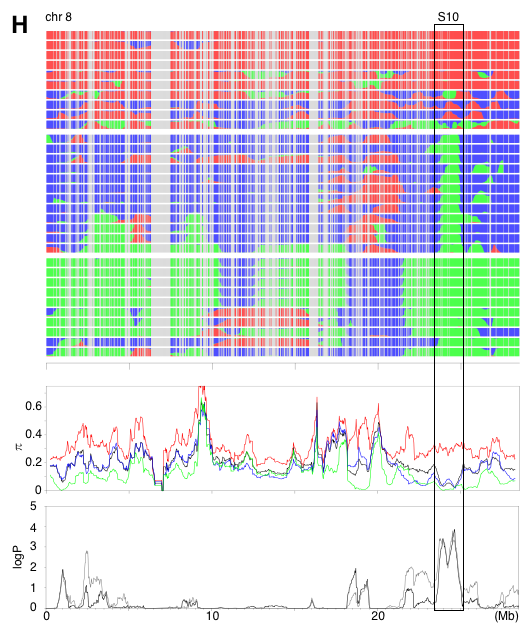

Supplement: Figure S3 — Genome-wide analysis of population structure and π for each chromosome. For each chromosome, the upper panel shows the results of STRUCTURE. Unaligned regions (mostly due to gaps) are in gray. The middle panel shows the genome wide distributions ofπ for each taxa. O. rufipogon is in red, indica in blue, japonica in green, and O. sativa (both indica and japonica included together) in black. The lower panel shows the statistical scores (logP) of the observed π (black) and θw (gray) of O. sativa/O. rufipogon, calculated by coalescent simulation [59]. The top 10 low diversity regions are indicated by black boxes (S1 to S10). The region that shows reduction of diversity specifically in indica is indicated by a blue box (I01). The regions that show exceptionally high FST between indica and japonica, and between tropical and temperate japonica are indicated by green boxes (IJ01 and JJ01, respectively). (ZIP) [file pone.0083720.s003.zip › FigureS3/chr08.png]

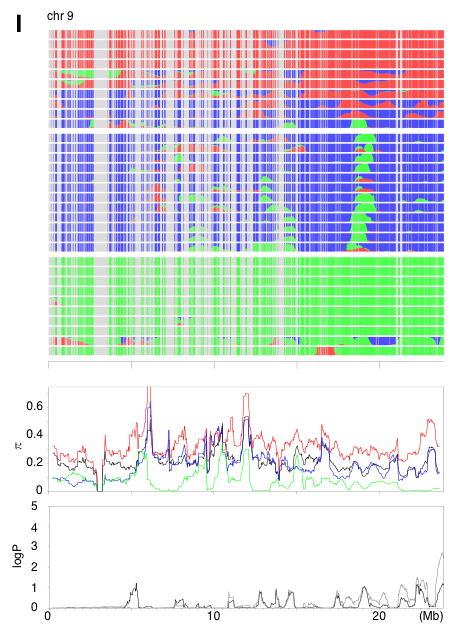

Supplement: Figure S3 — Genome-wide analysis of population structure and π for each chromosome. For each chromosome, the upper panel shows the results of STRUCTURE. Unaligned regions (mostly due to gaps) are in gray. The middle panel shows the genome wide distributions ofπ for each taxa. O. rufipogon is in red, indica in blue, japonica in green, and O. sativa (both indica and japonica included together) in black. The lower panel shows the statistical scores (logP) of the observed π (black) and θw (gray) of O. sativa/O. rufipogon, calculated by coalescent simulation [59]. The top 10 low diversity regions are indicated by black boxes (S1 to S10). The region that shows reduction of diversity specifically in indica is indicated by a blue box (I01). The regions that show exceptionally high FST between indica and japonica, and between tropical and temperate japonica are indicated by green boxes (IJ01 and JJ01, respectively). (ZIP) [file pone.0083720.s003.zip › FigureS3/chr09.png]

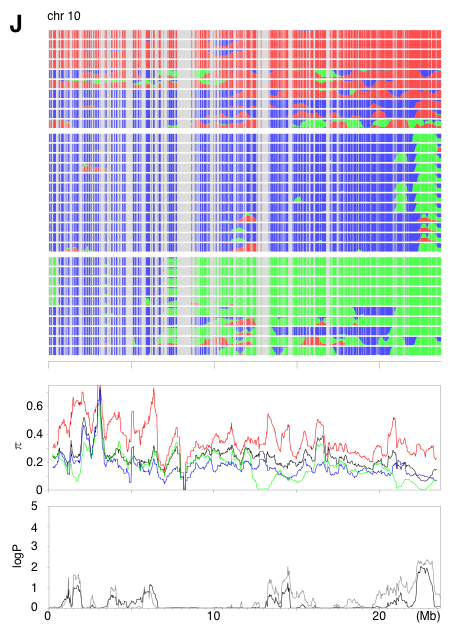

Supplement: Figure S3 — Genome-wide analysis of population structure and π for each chromosome. For each chromosome, the upper panel shows the results of STRUCTURE. Unaligned regions (mostly due to gaps) are in gray. The middle panel shows the genome wide distributions ofπ for each taxa. O. rufipogon is in red, indica in blue, japonica in green, and O. sativa (both indica and japonica included together) in black. The lower panel shows the statistical scores (logP) of the observed π (black) and θw (gray) of O. sativa/O. rufipogon, calculated by coalescent simulation [59]. The top 10 low diversity regions are indicated by black boxes (S1 to S10). The region that shows reduction of diversity specifically in indica is indicated by a blue box (I01). The regions that show exceptionally high FST between indica and japonica, and between tropical and temperate japonica are indicated by green boxes (IJ01 and JJ01, respectively). (ZIP) [file pone.0083720.s003.zip › FigureS3/chr10.png]

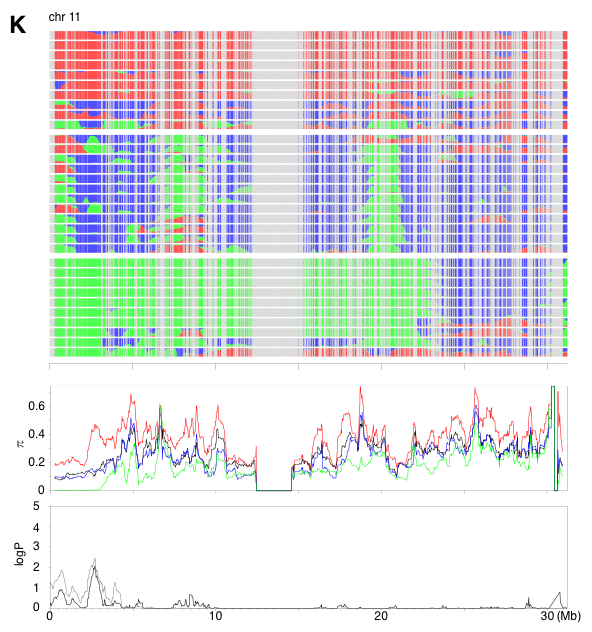

Supplement: Figure S3 — Genome-wide analysis of population structure and π for each chromosome. For each chromosome, the upper panel shows the results of STRUCTURE. Unaligned regions (mostly due to gaps) are in gray. The middle panel shows the genome wide distributions ofπ for each taxa. O. rufipogon is in red, indica in blue, japonica in green, and O. sativa (both indica and japonica included together) in black. The lower panel shows the statistical scores (logP) of the observed π (black) and θw (gray) of O. sativa/O. rufipogon, calculated by coalescent simulation [59]. The top 10 low diversity regions are indicated by black boxes (S1 to S10). The region that shows reduction of diversity specifically in indica is indicated by a blue box (I01). The regions that show exceptionally high FST between indica and japonica, and between tropical and temperate japonica are indicated by green boxes (IJ01 and JJ01, respectively). (ZIP) [file pone.0083720.s003.zip › FigureS3/chr11.png]

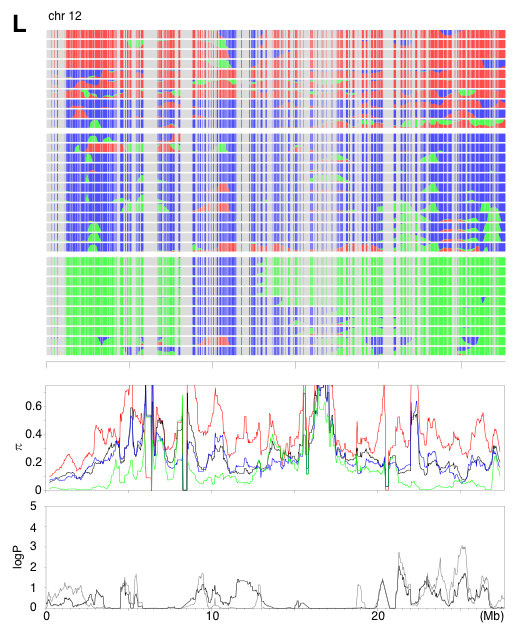

Supplement: Figure S3 — Genome-wide analysis of population structure and π for each chromosome. For each chromosome, the upper panel shows the results of STRUCTURE. Unaligned regions (mostly due to gaps) are in gray. The middle panel shows the genome wide distributions ofπ for each taxa. O. rufipogon is in red, indica in blue, japonica in green, and O. sativa (both indica and japonica included together) in black. The lower panel shows the statistical scores (logP) of the observed π (black) and θw (gray) of O. sativa/O. rufipogon, calculated by coalescent simulation [59]. The top 10 low diversity regions are indicated by black boxes (S1 to S10). The region that shows reduction of diversity specifically in indica is indicated by a blue box (I01). The regions that show exceptionally high FST between indica and japonica, and between tropical and temperate japonica are indicated by green boxes (IJ01 and JJ01, respectively). (ZIP) [file pone.0083720.s003.zip › FigureS3/chr12.png]
