# Supplementary material for: QTL Map Meets Population Genomics: An Application to Rice
Source: PLoS One. 2013 Dec 23;8(12):e83720. doi: 10.1371/journal.pone.0083720 (PMC3871663; doi:10.1371/journal.pone.0083720)
Supplement: Table S1 — Summary of sequencing. (DOCX) [file pone.0083720.s006.docx]

**Supplemental Table 1. Summary of sequencing.**

|  | |  | All mapped reads | | Reads with MapQ >50 | |  |  |
| --- | --- | --- | --- | --- | --- | --- | --- | --- |
|  | Accession | | Depth (fold) | Coverage  (%) | Depth (fold) | Coverage (%) | Number of SNPs^a^ | Number of Indels^a^ |
| 1 | W593 | | 30.7 | 80.6 | 27.4 | 68.8 | 2,258,815 | 364,450 |
| 2 | W1294 | | 18.8 | 80.5 | 16.9 | 69.2 | 2,126,551 | 318,261 |
| 3 | W1807 | | 16.5 | 80.0 | 14.7 | 68.7 | 2,034,517 | 304,430 |
| 4 | W2003 | | 17.6 | 80.1 | 15.6 | 68.6 | 2,139,884 | 320,060 |
| 5 | W1976 | | 19.5 | 79.4 | 17.6 | 67.3 | 2,152,181 | 335,324 |
| 6 | W2057 | | 22.0 | 78.3 | 19.8 | 66.3 | 2,049,375 | 322,400 |
| 7 | W0120 | | 19.5 | 81.3 | 17.7 | 69.7 | 1,981,703 | 307,196 |
| 8 | W630 | | 29.0 | 81.3 | 25.6 | 69.8 | 2,236,928 | 360,828 |
| 9 | W1866 | | 17.6 | 81.1 | 15.7 | 70.0 | 2,130,172 | 316,700 |
| 10 | W1965 | | 21.9 | 86.2 | 19.8 | 75.2 | 1,889,600 | 298,517 |
| 11 | BADARI DHAN (WRC39) | | 19.8 | 88.5 | 14.6 | 69.2 | 1,734,459 | 263,336 |
| 12 | KALUHEENATL (WRC41) | | 15.4 | 89.8 | 11.8 | 71.3 | 1,565,143 | 231,077 |
| 13 | KASALATH (WRC2) | | 12.4 | 86.5 | 8.8 | 67.5 | 1,502,060 | 222,690 |
| 14 | RATUL (WRC36) | | 19.3 | 88.5 | 13.6 | 69.1 | 1,642,055 | 262,577 |
| 15 | SHONI (WRC31) | | 17.4 | 86.8 | 12.3 | 67.6 | 1,485,284 | 245,737 |
| 16 | SURJAMUKHI (WRC33) | | 20.0 | 87.6 | 14.4 | 68.2 | 1,588,586 | 245,208 |
| 17 | TUPA121-3 (WRC32) | | 17.2 | 84.8 | 11.6 | 65.5 | 1,350,623 | 211,556 |
| 18 | JENA 035 (WRC4) | | 12.9 | 88.0 | 9.4 | 69.6 | 1,460,014 | 212,379 |
| 19 | DEEJIAOHUALUO (WRC98) | | 10.9 | 88.7 | 12.5 | 69.6 | 1,599,581 | 237,133 |
| 20 | HONG CHEUH ZAI (WRC99) | | 15.2 | 88.8 | 10.7 | 69.6 | 1,586,982 | 228,222 |
| 21 | KEIBOBA (WRC17) | | 12.5 | 86.8 | 8.8 | 68.1 | 1,528,699 | 216,803 |
| 22 | TAKANARI | | 10.7 | 88.6 | 7.7 | 69.9 | 1,303,925 | 182,486 |
| 23 | NIPPONBARE | | 25.0 | 98.7 | 18.0 | 85.5 | 8,998 | 4,044 |
| 24 | HITOMEBORE | | 35.1 | 97.8 | 27.6 | 84.3 | 106,230 | 28,606 |
| 25 | SASANISHIKI | | 17.7 | 95.8 | 13.8 | 81.5 | 77,433 | 20,031 |
| 26 | IWATEKKO | | 27.0 | 96.8 | 19.9 | 83.2 | 98,690 | 23,794 |
| 27 | DUNGHAN SHALI | | 6.0 | 92.3 | 4.4 | 76.6 | 151,458 | 25,661 |
| 28 | JAGUARY (WRC47) | | 15.7 | 94.7 | 12.6 | 79.4 | 432,908 | 77,540 |
| 29 | URASAN1 (WRC51) | | 16.7 | 93.1 | 13.0 | 77.4 | 545,360 | 93,769 |
| 30 | REXMONT (WRC50) | | 17.0 | 93.8 | 13.1 | 78.2 | 666,525 | 122,092 |
| 31 | TUPA 729 (WRC55) | | 16.8 | 91.1 | 13.0 | 74.1 | 976,866 | 153,607 |
| 32 | NERICA1 | | 13.5 | 92.2 | 10.7 | 76.0 | 762,596 | 120,517 |
|  | Wild rice | | 21.3 | 80.9 | 19.1 | 69.4 | 20,999,726 | 3,248,166 |
|  | Cultivated rice (excluding 32) | | 17.0 | 90.9 | 12.8 | 73.7 | 22,174,475 | 3,428,865 |
|  | Total | | 18.4 | 87.8 | 14.8 | 72.3 | 43,174,201 | 6,677,031 |

^a^ Numbers between each accession and the reference Nipponbare genome.
