# Supplementary material for: QTL Map Meets Population Genomics: An Application to Rice
Source: PLoS One. 2013 Dec 23;8(12):e83720. doi: 10.1371/journal.pone.0083720 (PMC3871663; doi:10.1371/journal.pone.0083720)
Supplement: Table S2 — Summary of SNPs and nucleotide diversity. (DOCX) [file pone.0083720.s007.docx]

|  |  | All (37,089,761 bp)^1^ | | |  | Synonymous  (2,695,798 bp)^1^ | | | | Replacement  (9,287,405 bp)^1^ | | | | Intron  (16,445,623 bp)^1^ | | |  | UTR (8,660,935 bp)^1^ | | |
| --- | --- | --- | --- | --- | --- | --- | --- | --- | --- | --- | --- | --- | --- | --- | --- | --- | --- | --- | --- | --- |
|  |  | SNPs | 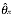^2^ | 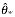^3^ |  | SNPs | 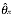^2^ | 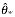^3^ |  | SNPs | 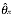^2^ | 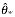^3^ |  | SNPs | 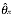^2^ | 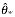^3^ |  | SNPs | 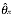^2^ | 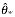^3^ |
| All | n=32 | 538,730 | 0.32 | 0.40 |  | 61,858 | 0.47 | 0.57 |  | 58,916 | 0.12 | 0.16 |  | 274,987 | 0.33 | 0.41 |  | 142,969 | 0.33 | 0.41 |
| *O. rufipogon* | n=10 | 434,129 | 0.41 | 0.32 |  | 50,736 | 0.60 | 0.46 |  | 46,409 | 0.15 | 0.12 |  | 221,772 | 0.42 | 0.33 |  | 115,212 | 0.41 | 0.33 |
| *O. sativa* | n=22 | 246,688 | 0.24 | 0.18 |  | 28,762 | 0.35 | 0.26 |  | 27,584 | 0.09 | 0.07 |  | 124,958 | 0.25 | 0.19 |  | 65,384 | 0.25 | 0.19 |
| *Indica* | n=12 | 185,008 | 0.20 | 0.14 |  | 21,869 | 0.29 | 0.20 |  | 20,532 | 0.08 | 0.05 |  | 93,022 | 0.20 | 0.14 |  | 49,585 | 0.20 | 0.14 |
| *Japonica* | n=10 | 114,528 | 0.11 | 0.09 |  | 13,621 | 0.16 | 0.12 |  | 13,049 | 0.04 | 0.03 |  | 57,786 | 0.11 | 0.09 |  | 30,072 | 0.11 | 0.09 |

**Supplemental Table 2. Summary of SNPs and nucleotide diversity.**

^1^Sites from 15,426 filtered set of genes.

^2^Estimator ofθ(4*Nμ*) based on the average numbers of pairwise nucleotide differences.

^3^Watterson’s estimator ofθ(4*Nμ*) based on the number of segregating sites.
