# Supplementary material for: QTL Map Meets Population Genomics: An Application to Rice
Source: PLoS One. 2013 Dec 23;8(12):e83720. doi: 10.1371/journal.pone.0083720 (PMC3871663; doi:10.1371/journal.pone.0083720)
Supplement: Text S1 — Supplementary methods. (DOCX) [file pone.0083720.s008.docx]

**Supplementary Methods**

**Screening SNPs**

The following screening process was applied to obtain high quality SNPs in single-copy genes (regions) as duplicated or repeated regions might cause confusion in identifying orthology. We first restricted our population genetic analyses to regions of protein-coding genes from the beginning of the 5’UTR to the end of the 3’UTR following the build 5 annotation of the Rice Annotation Project Database (http://rapdb.dna.affrc.go.jp). For these gene regions, further screening was carried out in order to reduce the number of unreliable SNP calls. First, regions surrounding microsatellites or indel polymorphisms were masked. Microsatellites with >9 mononucleotide-repeats, >5 dinucleotide-repeats, or >5 trinucleotide-repeats were excluded together with 8 bp of 5’ upstream and 12 bp of 3’ downstream regions. The 8 bp of 5’ upstream and 12 bp of 3’ downstream of positions where indel polymorphisms were called were also excluded. Sites containing ‘N’ in >5 out of 33 (including OM) accessions were also masked. As a consequence of these maskings, if a given region between masked regions (or between a masked region and the edge of the gene) ended up with ≤15bp, such a region was excluded.

Next, we examined sites that were identified as heterozygous. The heterozygosity is expected to be low as the species used here are selfing. Thus, regions containing an excess of heterozygosity may well be duplicated elsewhere within the genome. For rare heterozygous sites where only one out of 33 accessions were called as heterozygous and the other 32 homozygous, the heterozygous site was assigned the same nucleotide as the other homozygous sites assuming that such heterozygous sites are erroneous SNP calls. For the remaining heterozygous sites, we excluded genes if (number of heterozygous sites) / (gene region length excluding already masked nucleotides) > 0.001 in >3 accessions, or (number of sites that are heterozygous in >3 accessions) / (gene region length excluding already masked nucleotides) > 0.001. We then excluded genes if >30% of the total protein-coding sequences (CDS) were masked, or if the unmasked CDS was <150 bp. This screening process resulted in 1,042,719 SNPs in 31,036 genes. We estimated the rate of false positive SNP calls by resequencing a Nipponbare accession ourselves. By comparing this with the Nipponbare reference genome, we estimated the false positive rate to be 0.054% (excluding chromosome 4 that had an exceptionally large number of SNP calls). This is probably an overestimate because we are assuming that there are no sequencing errors in the reference genome and no genetic variations within the Nipponbare strain. Nevertheless, it is comparable to the rice resequencing study of [[1](#_ENREF_1)] (0.1%, although estimated in a different way), and more than an order lower than other resequencing studies (e.g. ~1.79% in the resequencing of soybean [[2](#_ENREF_2)]). The average number of pairwise nucleotide differences π for synonymous sites, nonsynonymous sites, UTRs, and introns were calculated also using a filtered dataset with genes containing in-frame stop codons and genes that do not begin with start codons or do not end with stop codons excluded. Although we do use SNPs within CDS or synonymous sites in some other analyses, these are merely to ensure that we are dealing with orthologous sites and thus should not actually matter whether they are coding or non-coding as long as the sites are orthologous.

**Coalescent simulations to test for selection**

In order to evaluate the local effect of selection, we performed a simulation-based test assuming a likely demographic scenario. We used a standard two-population model with a shared ancestral population (see Supplemental Figure 2A), which has been commonly applied to domesticated vs. progenitor species. We first focused on *O. rufipogon* vs *O. sativa*, where 12 *indica* and 10 *japonica* strains were pooled. We used 13,471 genes that contained 1,000 silent sites with reliable SNP data (see above). Only the first 1,000 sites were used to correct for the difference in length in each gene. Using these regions, we estimated the demographic parameters to summarize the bottleneck effect in the initial phases of the domestication process. Supplemental Figure 2A illustrates the model and parameters that were used. It is assumed that both populations are random-mating diploid populations. The population size of *O. ruﬁpogon* (*N_0_*) is assumed to be constant. Cultivated rice was domesticated from the wild progenitor *T_d_* generations ago and experienced a bottleneck event with a founder population with size *N_1_*. The period of the bottleneck was terminated *T_e_* generations ago, and then the population expanded to size *N_2_*, which is assumed to be identical to the current population size. The duration of bottleneck is defined as *T_1_* = *T_d_* − *T_e_*.

To reduce the labor of simulation, we fixed some parameters. We assumed *N_0_* = *N_2_*. This is because it is very difficult to estimate the current population sizes of cultivars as almost all samples are likely coalesced in the bottleneck phase. Furthermore, the effect of *N_2_* is known to be relatively minor [[3](#_ENREF_3),[4](#_ENREF_4),[5](#_ENREF_5)]. *T_d_* was fixed to be 10,000 generations ago based on the archaeological literatures, assuming one generation per year [[6](#_ENREF_6),[7](#_ENREF_7),[8](#_ENREF_8),[9](#_ENREF_9)]. In total, there are three parameters to be inferred, that is, *N_0_*, *N_1_* and *T_1_* (or *T_e_*).

We also fixed the rates of mutation and recombination. The mutation rate was fixed as µ = 10^-8^ [[10](#_ENREF_10),[11](#_ENREF_11)], where µ is the mutation rate per site per generation. The recombination rate was assumed to be constant across the genome at the rate of 4 cM/Mb [[12](#_ENREF_12)]. Finally, we introduced the algorithm of Nordborg and Donnelly [[13](#_ENREF_13)] for the coalescent process of selfing species because both wild and cultivated rice are selfing species. The recombination rate was also adjusted according to the selfing rate in each population [[14](#_ENREF_14)]. The selfing rate of cultivated rice was assumed to be 95% [[15](#_ENREF_15)]. In the *O. ruﬁpogon* population, annual and perennial plants have different selfing rates (roughly 95% and 40%, respectively) [[16](#_ENREF_16),[17](#_ENREF_17),[18](#_ENREF_18)]. Our sample is a mixture of annual (W630, W1866, W1807, W2003) and perennial plants (W593, W1294, W1976, W2057, W0120, W1965), and as such we used the weighted average (62 %).

With these fixed parameters, we estimated the demographic parameters, *N_0_*, *N_1_* and *T_1_* based on the observed numbers of segregating sites within the *O. rufipogon* and *O. sativa* samples, denoted by *S_r_* and *S_s_*, respectively. We first estimated the population size of *O. ruﬁpogon*, *N_0_*, alone from *S_r_* because in our model the likelihood of *S_r_* is given by a function of only *N_0_* and independent of *N_1_* and *T_1_*. We used the data of 13,471 genes with 1,000 silent sites and computed the log likelihood of the observed *S_r_*. This likelihood for the *i*th gene was denoted as *LL_i_*(*N_0_*|*S_r,i_*), where *S_r,i_* is the observed number of segregating sites in the *i*th gene in the *O. rufipogon* sample. Because it is very difficult to have an analytical expression for *LL_i_*(*N_0_*|*S_r,i_*) with recombination, we evaluated it by using a coalescent simulation. The simulation was performed by using the **ms** software (Hudson 2002) with a slight modification to allow changes in the selfing rate as described in [[5](#_ENREF_5)]. *LL_i_*(*N_0_*|*S_r,i_*) was obtained as the proportion of 10^8^ replications of simulation runs that has the exact same number of segregating sites, *S_r,i_*, denoted by *P_i_*(*S_r,i_*|*N_0_*). Then, the log likelihood for the data from all genes was computed as


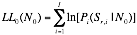
 (1)

where *I* is the number of genes (i.e., *I*=13,471). We computed *LL_0_*(*N_0_*) for *N_0_* = 150,000 ~ 250,000 at 5,000 intervals and we obtained *N_0_* = 185,000 as a maximum likelihood estimate. Because of the large number of genes analyzed simultaneously, this estimate has a very narrow 95% confidence interval (Supplemental Figure 2B), and thus, the following likelihood analysis was performed with *N_0_* fixed to this maximum likelihood estimate.

Next, we inferred *N_1_* and *T_1_*. The likelihood of the numbers of the segregating sites within *O .rufipogon* and within *O. sativa* (*S_r,i_* and *S_s,i_*, respectively) is given by a function of *N_1_* and *T_1_*. The likelihood for each gene, *P_i_*(*N_0_*,*T_1_*|*S_r,i_*,*S_s,i_*), was evaluated by 10^8^ replications of coalescent simulations, and the joint likelihood for all genes was computed as


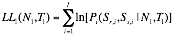
 (2)

We evaluated *LL_1_* for *N_1_* = 500 ~ 10, 000 and *T_1_* = 0 ~ 10, 000 at 500 intervals for both parameters, and the result is shown in Supplemental Figure 2C. We found the maximum likelihood estimates of *N_1_* = 7,000 and *T_1_* = 5,000.

This analysis can be applied to *indica* and *japonica* subspecies separately with *O. rufipogon* as the ancestor population. We obtained maximum likelihood estimates of (*N_1_*, *T_1_*) to be (3,500, 3000) and (7,000, 9,000) for *indica* and *japonica*, respectively.

It would be interesting to point out that according to previous reports [[4](#_ENREF_4),[19](#_ENREF_19)], it is thought that *N_1_* and *T_1_* are highly confounding and that these two parameters are indistinguishable. Therefore, to describe the severity of bottleneck, it was common to use *k* = *N_1_*/*T_1_*. Our results also show that they are highly correlated and parameter pairs with high likelihood distribute along a linear line, although there does seem to be a single peak. Our estimates are in good agreement with previous estimates in terms of *k*. Gao and Innan [[4](#_ENREF_4)] estimated *k* for *indica* and *japonica* to be 1.5 and 0.9, respectively, and *k* estimated in this study are 1.2 and 0.8.

**References**

1. Xu X, Liu X, Ge S, Jensen JD, Hu F, et al. (2012) Resequencing 50 accessions of cultivated and wild rice yields markers for identifying agronomically important genes. Nat Biotechnol 30: 105-111.

2. Lam H-M, Xu X, Liu X, Chen W, Yang G, et al. (2010) Resequencing of 31 wild and cultivated soybean genomes identifies patterns of genetic diversity and selection. Nat Genet 42: 1053-1059.

3. Eyre-Walker A, Gaut RL, Hilton H, Feldman DL, Gaut BS (1998) Investigation of the bottleneck leading to the domestication of maize. Proc Natl Acad Sci U S A 95: 4441-4446.

4. Gao L-z, Innan H (2008) Nonindependent domestication of the two rice subspecies, *Oryza sativa* ssp. *indica* and ssp. *japonica*, demonstrated by multilocus microsatellites. Genetics 179: 965-976.

5. Asano K, Yamasaki M, Takuno S, Miura K, Katagiri S, et al. (2011) Artificial selection for a green revolution gene during *japonica* rice domestication. Proc Natl Acad Sci U S A 108: 11034-11039.

6. Sweeney M, McCouch S (2007) The complex history of the domestication of rice. Ann Bot 100: 951-957.

7. Kovach MJ, Sweeney MT, McCouch SR (2007) New insights into the history of rice domestication. Trends Genet 23: 578-587.

8. Sang T, Ge S (2007) Genetics and phylogenetics of rice domestication. Curr Opin Genet Dev 17: 533-538.

9. Fuller DQ, Qin L, Zheng Y, Zhao Z, Chen X, et al. (2009) The domestication process and domestication rate in rice: spikelet bases from the Lower Yangtze. Science 323: 1607-1610.

10. Wolfe KH, Li WH, Sharp PM (1987) Rates of nucleotide substitution vary greatly among plant mitochondrial, chloroplast, and nuclear DNAs. Proc Natl Acad Sci U S A 84: 9054-9058.

11. Gaut BS, Clegg MT (1991) Molecular evolution of alcohol dehydrogenase 1 in members of the grass family. Proc Natl Acad Sci U S A 88: 2060-2064.

12. International Rice Genome Sequencing Project (2005) The map-based sequence of the rice genome. Nature 436: 793-800.

13. Nordborg M, Donnelly P (1997) The coalescent process with selfing. Genetics 146: 1185-1195.

14. Nordborg M (2000) Linkage disequilibrium, gene trees and selfing: an ancestral recombination graph with partial self-fertilization. Genetics 154: 923-929.

15. Oka HI (1988) Origin of Cultivated Rice: Japan Sci. Soc. Press/Elsevier, Tokyo/Amsterdam.

16. Oka HI, Morishima H (1967) Variations in the breeding systems of a wild rice, *Oryza perennis*. Evolution 21: 249-258.

17. Barbier P (1989) Genetic variation and ecotypic differentiation in the wild rice *Oryza rufipogon* II. Influence of the mating system and life history traits on the genetic structure of populations. Jpn J Genet 64: 273-285.

18. Morishima H, Barbier P (1990) Mating system and genetic structure of natural populations of wild rice *Oryza rufipogon*. Plant Species Biol 5: 31-39.

19. Wright SI, Bi IV, Schroeder SG, Yamasaki M, Doebley JF, et al. (2005) The effects of artificial selection on the maize genome. Science 308: 1310-1314.
